# Topological Control of Dual Protonic–Electronic Conduction in Metal–Organic Frameworks

**DOI:** 10.1021/jacs.6c05964

**Published:** 2026-07-02

**Authors:** Huilin Qing, Priyanshu Chandra, Joseph Y. M. Chan, Richard J. Staples, Tai-De Li, Weiyang Li, Katherine A. Mirica

**Affiliations:** † Thayer School of Engineering, 3728 Dartmouth College, Hanover 03755, New Hampshire, United States; ‡ Department of Chemistry, 3728 Dartmouth College, Hanover 03755, New Hampshire, United States; § Department of Chemistry, 3078 Michigan State University, East Lansing 48824, Michigan, United States; ∥ Nanoscience Initiative, Advanced Science Research Center, CUNY Graduate Center, City University of New York, New York 10031, New York, United States; ⊥ Department of Physics, City College of New York, City University of New York, New York 10031, New York, United States

## Abstract

Metal–organic frameworks (MOFs) with intrinsic dual proton–electron conductivity are highly desirable for energy conversion devices and chemical separation, yet merging these properties within a single crystalline phase remains a challenge. Here, we report two novel Mn­(II)-based conjugated MOFs that share similar building blocks, but diverge into distinct topologies: a kagome lattice (**kgm**) and an unprecedented pseudo **bex-d** topology (Mn-HHTP-**bex-d**). These frameworks exhibit sharply contrasting conduction profile: Mn-HHTP*-* **kgm** demonstrates excellent electronic conductivity (8.4 × 10^–1^ S cm^–1^ at room temperature), but limited proton transport (3.6 × 10^–7^ S cm^–1^) at 98% relative humidity (RH), whereas the pseudo **bex-d** topology exhibits more balanced electronic conductivity (2.4 × 10^–5^ S cm^–1^) and proton conductivity (4.5 × 10^–5^ S cm^–1^ at 98% RH). Crystallographic and computational studies indicate that the efficient π–π stacking in **kgm** topology promotes charge delocalization and through-space charge transport for electrical conduction, while the pseudo **bex-d** topology leverages framework-incorporated water molecules and acetate moieties to establish efficient hydrogen-bonding networks for proton transport. This work highlights the critical role of topological control in modulating mixed-conduction properties and offers valuable insights for designing multifunctional MOFs for ambipolar devices, bioelectronics, and energy systems.

## Graphical abstract

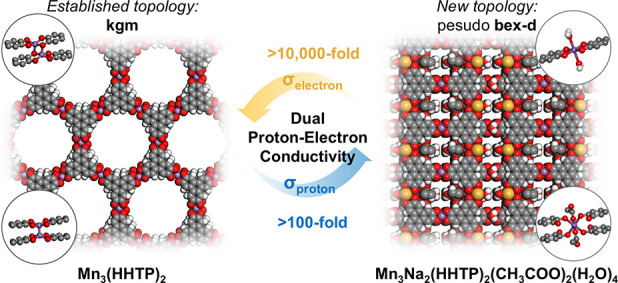

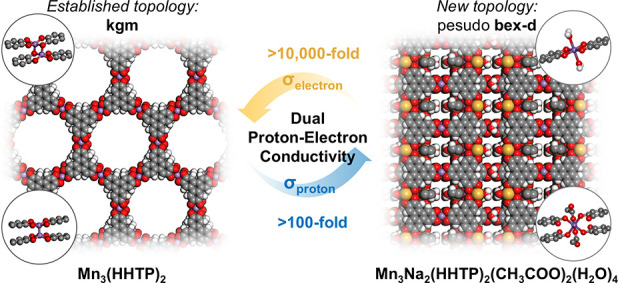

## Introduction

The rising demand for materials innovation to support the development of advanced technologies, such as fuel cells, supercapacitors, gas sensors, actuators, and chemical separation membranes, has highlighted the significance of mixed proton–electron conductors over the past several decades.^1–6^ Such implications arise from the essence that several chemical/biological processes and electrochemical reactions (e.g., photosynthesis, enzymatic reactions, electrocatalytic CO_2_ reduction, etc.) necessitate both proton and electron transfer and the awareness of the significance of proton-coupled electron transfer (PCET) steps.^7,8^ Materials with dual protonic–electronic conductivity are pivotal in enhancing efficiency and maximizing energy conversion performance of these devices by enabling simultaneous and rapid mass and charge transport. Traditionally, dual proton–electron conductors have been fabricated from composites through physical blending. The resulting composites, however, suffer from poorly defined structure and disordered phase distributions, resulting in uncontrollable conducting pathways and inefficient transmission efficiency.^2,9^ For example, graphene oxide and perovskite composites have shown excellent properties, yet challenges persist in establishing certain structure–property relationships and elucidating the underlying transport mechanism, primarily due to their structural ambiguity.^10,11^

To overcome these limitations, developing single-phase materials with well-defined crystal structures that support both intrinsic electronic and protonic conduction is of significant interest. In this regard, metal–organic frameworks (MOFs) have emerged as promising candidates as their regular periodic and tunable structures enable systematic investigations of conduction mechanisms and deliberate modulation in either electron or proton conduction processes.^7,12^ Electronic conductivity in MOFs is generally achieved through the incorporation of extended π-conjugated ligands, such as triphenylene derivatives, which facilitate electron delocalization via *d*−π orbital overlaps and π–π stacking interactions.^13–16^ However, the limited coordination geometries of metal nodes and the scarcity of functionalizable sites on fully conjugated linkers constrain the diversity of conductive MOF topologies and functionalities.^17–19^ Proton conductivity, on the other hand, is typically enhanced by introducing hydrophilic functional groups or molecules to promote hydrogen-bonding networks for proton transfer.^20–23^ Thus, integrating both protonic and electronic transport pathways in a single MOF has remained challenging due to the seemingly conflicting requirements in molecular design—namely, the difficulty in functionalizing extended π-conjugated ligands without compromising electronic delocalization. These divergent design strategies have historically led to parallel, rather than convergent, development of proton- and electron-conductive MOFs.

Several alternative approaches have been proposed to integrate proton conductivity into electrically conductive MOFs. Examples include postsynthetic modification with urea/phosphoric acid,^3,22^ designing conjugated organic linkers rich in B, N, S, P-heteroatoms,^17,24–26^ and utilizing high-coordination-number metal centers to accommodate multifunctional groups or molecules.^3,26–28^ Nevertheless, only a handful of dual-conductive MOFs have been reported in the past five years, and their proton-conducting pathways remain ambiguous.^1,3,12,24–26,29^ Therefore, the discovery and thorough characterization of structurally well-defined MOFs capable of simultaneous proton and electron conduction are imperative for advancing our fundamental understanding of structure–conduction relationships and unlocking their full potential in advanced technologies.

In this context, we report two new MOF structures constructed from Mn^2+^ nodes and triphenylene-based ligands, specifically 2,3,6,7,10,11-hexahydroxytriphenylene (HHTP). The resulting two frameworks, herein referred to as Mn-HHTP-**kgm** and Mn-HHTP-**bex-d**, adopt distinct structural topologies: Mn-HHTP-**kgm** in a formula of Mn_3_(HHTP)_2_ features the characteristic kagome lattice and honeycomb motif typical of serrated π-stacked triphenylene-based MOFs, while Mn-HHTP-**bex-d** shows a structural formula of Mn_3_Na_2_(HHTP)_2_(CH_3_COO)_2_(H_2_O)_4_ and forms an unprecedented pseudo **bex-d** topology comprising dual layers (Figure 1). We focus on the structural elucidation and structural topology-induced differences in conduction properties between these two MOFs. Remarkably, Mn-HHTP-**kgm** exhibits a significantly enhanced electronic conductivity of 8.4 × 10^–1^ S cm^–1^ than the pseudo **bex-d** topology (2.4 × 10^–5^ S cm^–1^) at room temperature, attributed to its favorable π–π stacking interactions and shorter interlayer distances. In contrast, Mn-HHTP-**bex-d** demonstrates superior proton conductivity of 4.5 × 10^–5^ S cm^–1^ at 298 K and 98% relative humidity (RH), approximately 2 orders of magnitude greater than that of Mn-HHTP-**kgm** (3.6 × 10^–7^ S cm^–1^), owing to its increased water coordination and acetate ions for hydrogen-bonding network. This work contributes to the growing field of dual-conductive MOFs and offers new insights into structure-driven transport mechanisms, which are essential for the rational design of next-generation multifunctional and diverse MOF materials.

**Figure 1:**
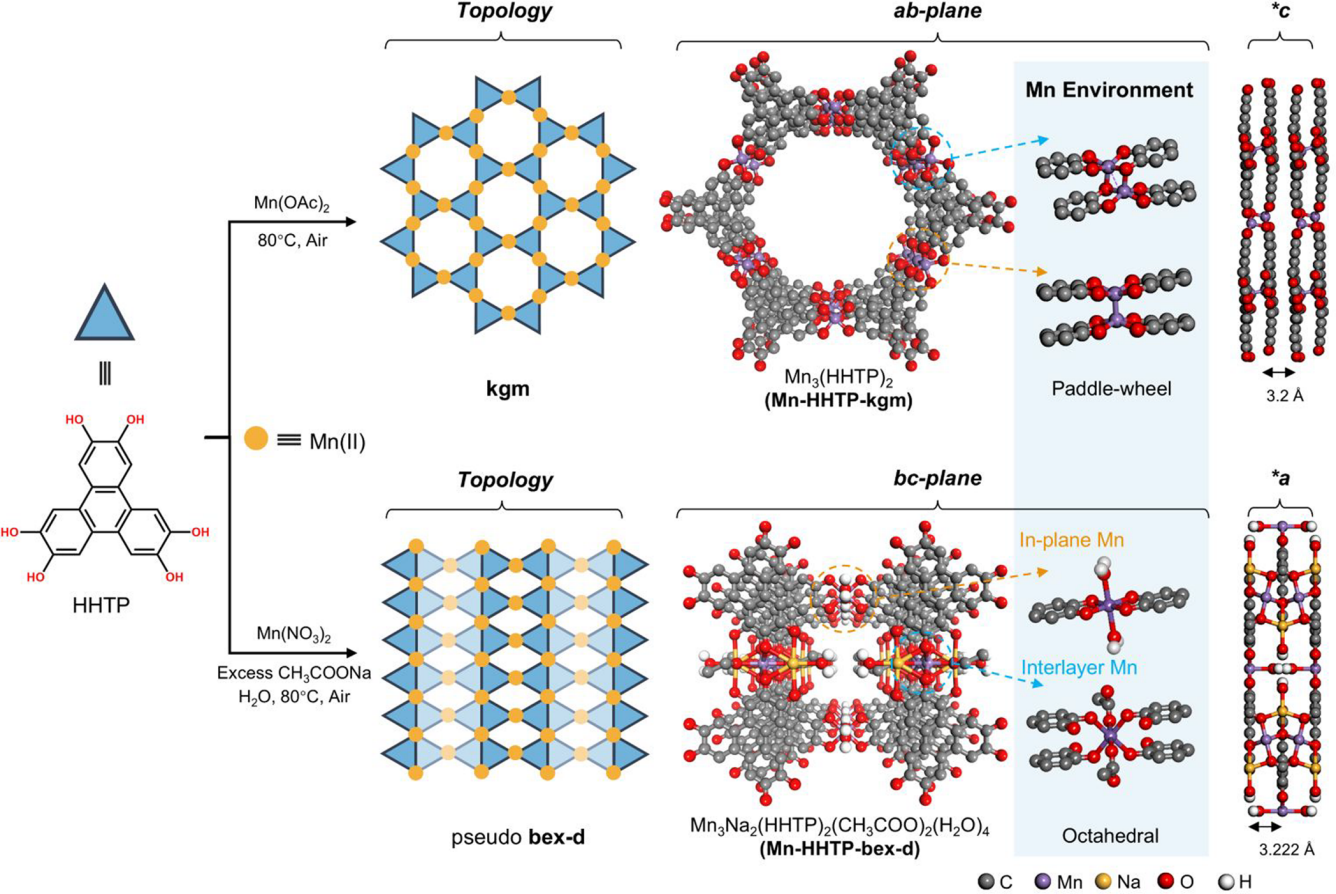
Synthetic schemes, topologies, in-plane structures and out-of-plane structures of Mn_3_(HHTP)_2_ MOF (Mn-HHTP-**kgm**) and Mn_3_Na_2_(HHTP)_2_(CH_3_COO)_2_(H_2_O)_4_ MOF (Mn-HHTP-**bex-d**). The H atoms in the HHTP ligands and acetate groups are omitted to clearly show the in-plane structure of the two materials. One representative configuration of the disordered methyl groups from the acetates in Mn-HHTP-**bex-d** is shown for clarity.

## Results and Discussion

### Synthesis and Crystal Structure

Mn-HHTP-**kgm** and Mn-HHTP-**bex-d** were both synthesized via hydrothermal methods using Mn­(II) metal salts and HHTP organic linkers (Figure 1). The key to dictating the formation of distinct packing structures was the amount of sodium acetate used during synthesis. With no sodium acetate or even a small amount, only Mn-HHTP-**kgm** was obtained. In contrast, with an excess amount of sodium acetate at a molar ratio of 60:1 relative to the HHTP ligand, Mn-HHTP-**bex-d** was formed. While sodium acetate was commonly used to adjust pH and control nucleation kinetics in MOF synthesis,^30,31^ its role here was crucial in directing and stabilizing the pseudo **bex-d**-type topology. Although the crystallization mechanism in solution remains elusive, one possible explanation based on Hard and Soft Acid and Base (HSAB) theory is that slightly different from borderline acids (i.e., Cu^2+^, Ni^2+^, Co^2+^, and Zn^2+^), Mn^2+^, as a hard acid, can interact with hard bases (i.e., acetate ions) more favorably and stably.^32–35^ Excess sodium acetate intensified the competition between acetate ions and HHTP ligands for interaction with Mn^2+^, thereby initiating the construction of a more complex pseudo **bex-d** topology. We investigated a series of synthetic parameters, including concentration, solvent, and oxygen content, to obtain a highly crystalline structure for further investigation (Table S1 and Figure S1). For Mn-HHTP-**kgm**, the content of oxygen to promote partial oxidation of HHTP linker played an important role in crystal growth and quality, which is akin to observations in other triphenylene-based MOFs (e.g., Cu_3_(HHTP)_2_ and Co_3_(HITP)_2_).^36–39^ For Mn-HHTP-**bex-d**, ensuring sufficient premixing of HHTP linkers and Mn­(NO_3_)_2_ prior to the addition of sodium acetate allowed for uniform coordination and growth of high-quality single crystals.

To elucidate the structures of the as-synthesized MOFs, we initially collected powder X-ray diffraction (PXRD) patterns from polycrystalline samples. Mn-HHTP-**kgm** exhibited diffraction patterns closely matching those of previously reported 2D M_3_(HHTP)_2_ (M = Cu, Ni, Co) MOFs^40^ and was best modeled by a serrated-parallel AA′ stacking structure^41,42^ with a residue (*R*_wp_) of 0.53% (Figures 1 and S2–S6; Table S2). PXRD reflections at 2θ = 4.74°, 9.51°, and 12.61° were indexed to the (100), (200) and (3 $\overset{-}{1}$ 0) planes of the simulated two-dimensional (2D) honeycomb framework (Figure 2a). The broad peak at 2θ = 28.3° corresponded to overlapping contributions from the higher-index planes and the (102) and (002) planes, closely related to the stacking spacing. The structural optimization began with a serrated-parallel AA′ stacking structure, in which each Mn­(II) center adopted a square planar coordination geometry. This starting configuration was inspired by the Cu_3_(HHTP)_2_ structure and by reported examples of square-planar Mn­(II) coordination in MOFs and molecular complexes where Mn­(II) binds to planar, highly conjugated ligands. For instance, Long et al. reported a square-planar geometry of Mn­(II) in the Mn_3_(C_6_S_6_) conjugated MOF with an eclipsed stacking mode;^43^ similarly, the square-planar environment of the Mn center in manganese­(II) phthalocyanine is well-established.^44,45^ Upon structural optimization with a van der Waals correction, the simulated serrated-parallel AA’ stacking structure showed that the Mn atoms were squeezed into short Mn–Mn contacts (<3 Å) (Figures 1 and S5). As a result, the coordination environment of these Mn centers became distorted into a square pyramidal geometry, with the apical position occupied by either an O or a Mn atom. These dimeric Mn­(II) centers formed a paddle-wheel motif with four HHTP molecules. Although the short metal–metal contacts remain uncommon in the 2D layered conjugated MOFs, binuclear or multicentered metal–metal interactions have been reported to possibly exist in organometallics^46–48^ and reticular framework systems^49^ as a consequence of the ability of metal centers to adopt complicated coordination geometries, the rigidity of the ligand, and steric demand. The dimeric Mn­(II) paddle-wheel centers in Mn-HHTP-**kgm** align well with the computed structure of Mn_2_(PhCOO)_4_ in Mn-BTC MOF^50^ and the experimental structure of Mn_2_(PhCOO)_4_Cl_2_ nodes in Mn-TCPP­(Ru) MOF.^51^ Moreover, the recent precedent describing interlayer metalloid Ge–Ge bonding in analogous framework material^52^ may suggest that these interlayer Mn–Mn contacts are chemically viable. The possible formation of interlayer Mn–Mn close contacts is in agreement with the notably short interlayer spacing (3.2 Å) in the structure. Despite some structural insights gained from computational simulations, literature review, and preliminary efforts at structural determination using micro electron diffraction (Figure S7), it should be noted that the exact crystal structure of Mn-HHTP-**kgm** will need to be unambiguously resolved by definitive crystallographic determinations in future studies.

**Figure 2:**
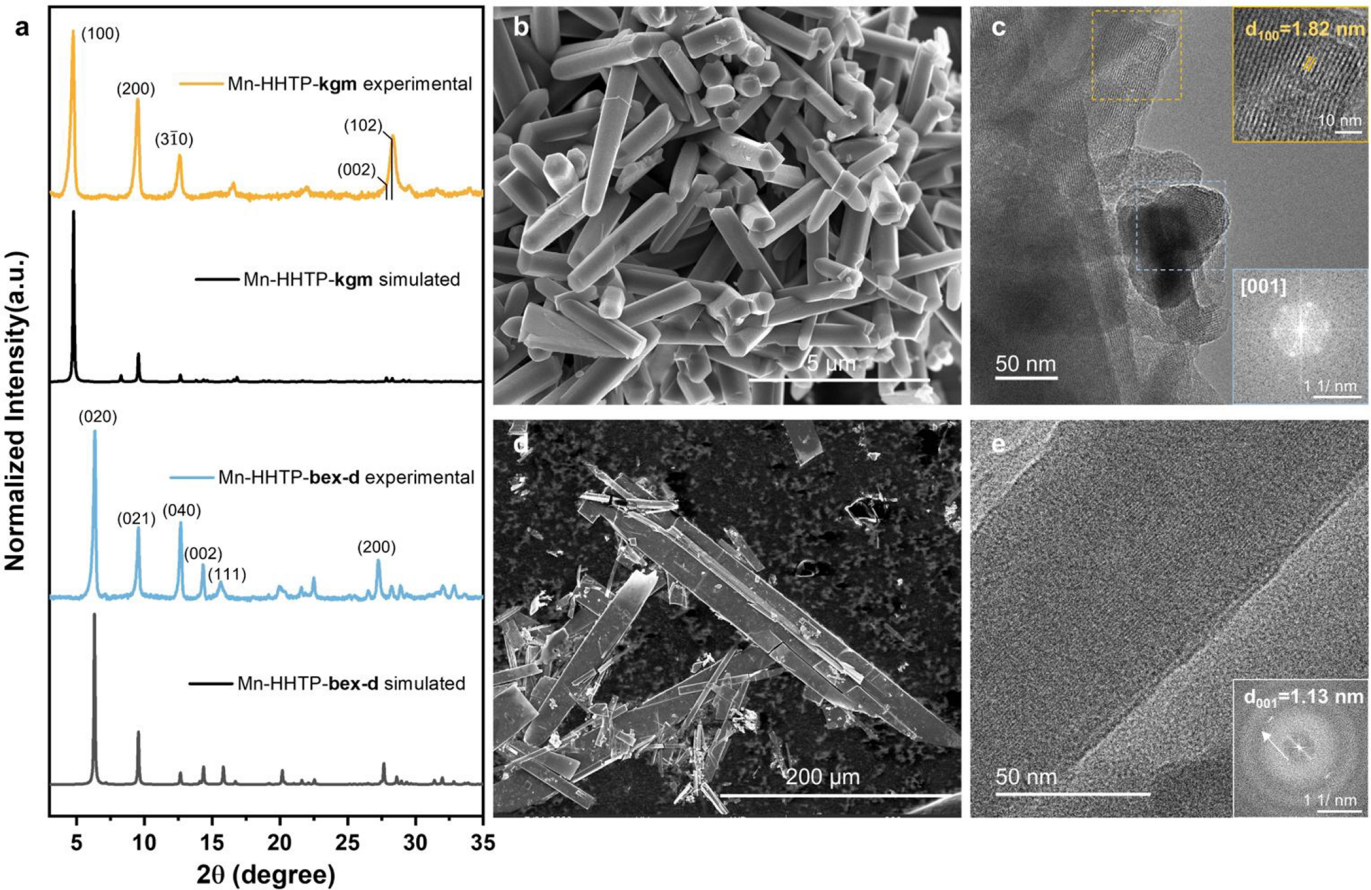
(a) PXRD of Mn-HHTP-**kgm** and Mn-HHTP-**bex-d** MOFs after background subtraction. (b) SEM image of Mn-HHTP-**kgm**. (c) TEM image of Mn-HHTP-**kgm**. Inset: Zoom-in image of selected area (top) and FFT pattern of selected area (bottom). (d) SEM image of Mn-HHTP-**bex-d.** (e) TEM image of Mn-HHTP-**bex-d**. Inset: corresponding FFT pattern.

Mn-HHTP-**bex-d** exhibited an entirely different PXRD pattern. Its detailed crystal structure was further determined by single-crystal XRD (SCXRD) measurement on a suitable Mn-HHTP-**bex-d** crystal with dimensions of 0.21 × 0.07 × 0.04 mm^3^ (Figure S8). Despite the presence of disordered methyl groups and water molecules, the main framework remained well resolved (Figures 1 and S9–S11). The analysis revealed that Mn-HHTP-**bex-d** crystallized in the orthorhombic space group *Cmmm* with lattice parameters of *a* = 6.44 Å, *b* = 27.95 Å, *c* = 12.32 Å to yield the structural formula as Mn_3_Na_2_(HHTP)_2_(CH_3_COO)_2_(H_2_O)_4_ (Table S3). In the resolved single-crystal structure, parallel π–π stacking of the organic HHTP ligands was observed along the *a*–axis direction, indicating a pseudo-2D layered structure. PXRD of Mn-HHTP-**bex-d** at 2θ = ∼ 6.35°, 9.56°, 12.7°, 14.3°, 15.6° and 27.26° corresponded to its (020), (021), (040), (002), (111), and (200) planes (Figure 2a). Detailed analysis of the experimentally obtained PXRD patterns (Figure S12) showed that the interlayer stacking distance in Mn-HHTP-**bex-d** was greater than that of Mn-HHTP-**kgm**, consistent with the values from the simulated structure of Mn-HHTP-**kgm** (3.2 Å) and single-crystal structure of Mn-HHTP-**bex-d** (3.222 Å). Unlike Mn-HHTP-**kgm**, Mn-HHTP-**bex-d** contains two distinct types of Mn­(II) centers with octahedral coordination geometry. One exhibits bidentate chelation with two different HHTP linkers in the equatorial plane and coordination with two axial water molecules, designated as the in-plane Mn center. The other Mn center, denoted as interlayer Mn, is located between the two HHTP layers and is bonded to four oxygen atoms from four different HHTP linkers and two oxygen atoms from two acetate groups. The in-plane and interlayer Mn nodes are 2-connected and 4-connected, respectively. Due to the existence of interlayer Mn centers, the connectivity of the HHTP ligand switches from 3 in **kgm** to 5 in this topology. Additionally, Na^+^ ions are incorporated into the framework and exhibit a distorted pentagonal bipyramidal coordination geometry. These unique coordination environments induce the framework structure adopting an ABAB layered 3D stacking pattern with an interlayer distance of 3.222 Å, featuring well-defined rectangular pores.

From a topological perspective, Mn-HHTP-**kgm** adopts a kagome lattice (**kgm**), while Mn-HHTP-**bex-d** conforms to a new topology that can be described as a (2,4,5)-connected net with both Mn and HHTP ligand treated as central groups (Figures 1, S13, and S14). Notably, when only Mn atoms are considered as the central nodes, the resulting net of Mn-HHTP-**bex-d** closely resembles the theoretically proposed 2D **bex-d** topology (Figure S15), which has not yet been experimentally established in MOF systems. Accordingly, we denoted this newly observed topology within the rectangular framework as a pseudo **bex-d** topology. In the ideal 2D monolayer **bex-d** topology, the network consists of a 2-nodel net with 4, 6-connected nodes. By contrast, the observed pattern arises from geometric overlap between adjacent layers in a pseudo-2D bilayer system, where the extension in dimensionality increases the net connectivity to 8 and 12. Rectangular frameworks based on such pseudo **bex-d** topology are rarely encountered in reticular chemistry and, to the best of our knowledge, are unprecedented among electrically conductive MOFs. Although MOFs are renowned for their structural diversity and complexity in reticular design, the accessible topological space of layered conjugated MOFs with intrinsic electronic conductivity has remained relatively narrow. The prevalent topologies in this domain have predominantly been limited to hexagonal, kagome, square, and rhombic networks, typically arising from organic linkers with C_6_, C_3_, C_4_, and D_2_ symmetries, respectively (Figure S16).^15,16^ Particularly in layered MOFs, the commonly employed transition metal nodes (i.e., Cu, Co, Ni) preferentially coordinate with bidentate chelating groups on ligands (e.g., *ortho*-substituted −NH_2_, −OH, or −SH) in a saturated square-planar or equatorial octahedral geometry with a limited connectivity, which in turn constrains topological diversity and structural complexity. The realization of a pseudo **bex-d** topology from C_3_-symmetric linkers in layered MOFs therefore represents a significant expansion of the topological landscape and offers new opportunities to explore structure–property relationships in reticular frameworks.

The distinct structural topologies were validated by the morphological imaging. Scanning electron microscopy (SEM) image in Figure 2b revealed hexagonal rod-shaped crystallites of Mn-HHTP-**kgm** with lengths in submicrons, whereas Mn-HHTP-**bex-d** crystallized into larger cuboidal-/plate-like crystals exceeding 200 μm in length (Figure 2d). The energy-dispersive X-ray spectroscopy (EDS) spectra confirmed the presence of Mn, C and O elements and their uniform distribution in Mn-HHTP-**kgm** (Figures S17 and S18), while the EDS spectra for Mn-HHTP-**bex-d** revealed the additional presence of Na (Figures S19 and S20). Transmission electron microscopy (TEM) imaging perpendicular to the long axis of Mn-HHTP-**kgm** displayed single-crystalline lattice patterns with periodicity of 1.82 nm corresponding to the *d*_100_ in the proposed structure (Figure 2d). Micrographs exploring the in-plane arrangement of facing-up Mn-HHTP-**kgm** particles presented a well-resolved honeycomb framework with a 6-fold symmetric dot matrix in the fast Fourier transform (FFT) image featuring a hexagonal pore (Figures 2c and S21). In Mn-HHTP-**bex-d**, parallel lattice fringes were observed with a measured interlayer spacing of 1.13 nm, slightly lower than the calculated 1.23 nm *d*_001_ value (Figure 2e). The slight mismatch was due to the beam-sensitive nature of Mn-HHTP-**bex-d**, whose Bragg plane contracted under high-energy electron beam, consistent with the behavior observed in some MOF-based materials.^53–55^ Furthermore, low-temperature (77 K) N_2_ sorption measurement for Mn-HHTP-**kgm** revealed a superposition of type I and type II with a rapid increase in nitrogen adsorption at low pressures indicative of microporosity, and it exhibited a Brunauer–Emmett–Teller (BET) surface area of 216 m^2^/g (Figure S22). Conversely, Mn-HHTP-**bex-d** showed a significantly lower BET surface area of 28 m^2^/g. Compared with the pseudo **bex-d** network, the **kgm** framework has larger pores of ∼2 nm (Figure S23). The reduced porosity of Mn-HHTP-**bex-d** might be attributed to the smaller pore size from the pseudo **bex-d** topology and the occupation of water molecules and sodium acetate in the pores of Mn-HHTP-**bex-d**.

Further structural analysis on the two different MOFs via electron paramagnetic resonance (EPR) at room temperature revealed that both structures had similar *g*-factors (2.008 for Mn-HHTP-**kgm** and 2.005 for Mn-HHTP-**bex-d**), indicating that the Mn ions in each structure were in the same oxidation state, likely Mn­(II) (Figure S24). X-ray photoelectron spectroscopy (XPS) confirmed the presence of C, O, and Mn in Mn-HHTP-**kgm** and the presence of C, O, Mn, Na in Mn-HHTP-**bex-d** (Figures S25 and S26). The high-resolution Mn 3*s* photoemission spectra disclosed the predominant presence of manganese species in the +2 state with a peak splitting energy of 6.0 eV.^56^ These results confirmed a similar electronic environment and the uniform oxidation state of Mn across both structures. Moreover, the peak at ∼535.5 eV in O 1s spectrum corresponded to the H_2_O species coordinated/adsorbed in Mn-HHTP-**bex-d**, and the enhanced signals for C=O in both C 1*s* and O 1*s* spectra of Mn-HHTP-**bex-d** indicated the presence of acetate ions. This was also evident in the Fourier-transform infrared (FT-IR) spectrum of Mn-HHTP-**bex-d**, where characteristic symmetric and asymmetric stretching modes of carboxylate groups (COO^–^) appeared near 1407 and 1565 cm^–1^, respectively (Figure S27).

We also evaluated the structural stabilities of the two MOFs with different topologies. Mn-HHTP-**kgm** maintained crystallinity for at least two months under ambient conditions (room temperature and dry air) (Figure S28) and exhibited thermal stability up to 200 °C in air, as confirmed by thermogravimetric analysis (TGA) (Figure S29). However, Mn-HHTP-**bex-d** exhibited continuous weight loss upon heating in air (Figure S29) and rapidly lost crystallinity (Figure S30). We reasoned the loss of crystallinity for the removal of water molecules, given the essence of structural water molecules to framework integrity.^57^ To preserve its structure, Mn-HHTP-**bex-d** was stored in a freezer, which significantly improved the stability of Mn-HHTP-**bex-d**. Additionally, TGA analysis demonstrated a higher water content in Mn-HHTP-**bex-d** than Mn-HHTP-**kgm**. The above results validated the successful assembly of Mn­(II) and HHTP ligands into distinct framework structures, consequently correlating with different properties.

### Electronic Structure and Electronic Conductivity

Following the structural elucidation of Mn-HHTP-**kgm** and Mn-HHTP-**bex-d**, we investigated their electronic properties, hypothesizing that the distinct topologies of these MOFs would significantly alter their electronic structure. We performed density functional theory (DFT) calculations to obtain the band structures and projected density of states (PDOS) of both materials (see Supporting Information for details). Optimized structures of both MOFs were employed for energy calculation (Figures S2 and S31). Given their layered features, high-symmetry points in the first Brillouin zones were sampled to cover both in-plane (*ab*-plane for Mn-HHTP-**kgm** and *bc*-plane for Mn-HHTP-**bex-d**) and out-of-plane (*c*-axis for Mn-HHTP-**kgm** and *a*-axis for Mn-HHTP-**bex-d**) directions (Figures 3a, and S32–S33). Mn-HHTP-**kgm** displayed relatively flat bands along the in-plane Γ–M–K pathway with a small bandgap of ∼0.17 eV, suggesting semiconducting character in the plane, while there is pronounced band dispersion along the Γ–A path (*c*-direction), where the conduction band crossed the Fermi level (*E*_F_), indicative of metallic behavior along the stacking direction (Figure 3b). The PDOS revealed that states near the Fermi level were primarily derived from the C and O valence orbitals of the HHTP linkers, with negligible contributions from the highly ionic Mn­(II) centers. This result supports a through-space electron transport mechanism via π–π stacking between adjacent linkers. In comparison, Mn-HHTP-**bex-d** band structure exhibited metallic features along both in-plane (Γ–Z–T−Γ) and out-of-plane (Γ–X) pathways (Figure 3f). The through-space charge-transport might still contribute significantly to electron conduction in Mn-HHTP-**bex-d**, as suggested by the larger band dispersion along the Γ–X pathway relative to the in-plane directions as well as the dominant contributions of the HHTP ligands and interlayer Mn centers in the PDOS (Figure S34). However, compared with the serrated-parallel AA′ stacking in Mn-HHTP-**kgm**, noticeable distortion and mismatch in π–π stacking of layered Mn-HHTP-**bex-d** (Figures 3c,g) and increased stacking distance lowered the through-space conduction efficiency, which was evidenced by the reduced band curvature along the out-of-plane pathway (maximum band dispersions crossing *E*_F_ of 525 and 327 meV for Mn-HHTP-**kgm** and Mn-HHTP-**bex-d**, respectively). Consistently, electron density difference maps along the out-of-plane direction (Figures 3d,h) reveal more extensive charge delocalization in Mn-HHTP-**kgm** than in the pseudo **bex-d** topology, supporting more efficient through-space conduction in Mn-HHTP-**kgm**. Mn-based orbitals contributed slightly more near the Fermi level in Mn-HHTP-**bex-d** than in Mn-HHTP-**kgm**, suggesting a minor role of Mn centers in modulating the electronic states (Figure 3f). The close-to-zero population of Na species near the Fermi level suggested the neglectable effects of Na^+^ incorporation on electronic structures.

**Figure 3:**
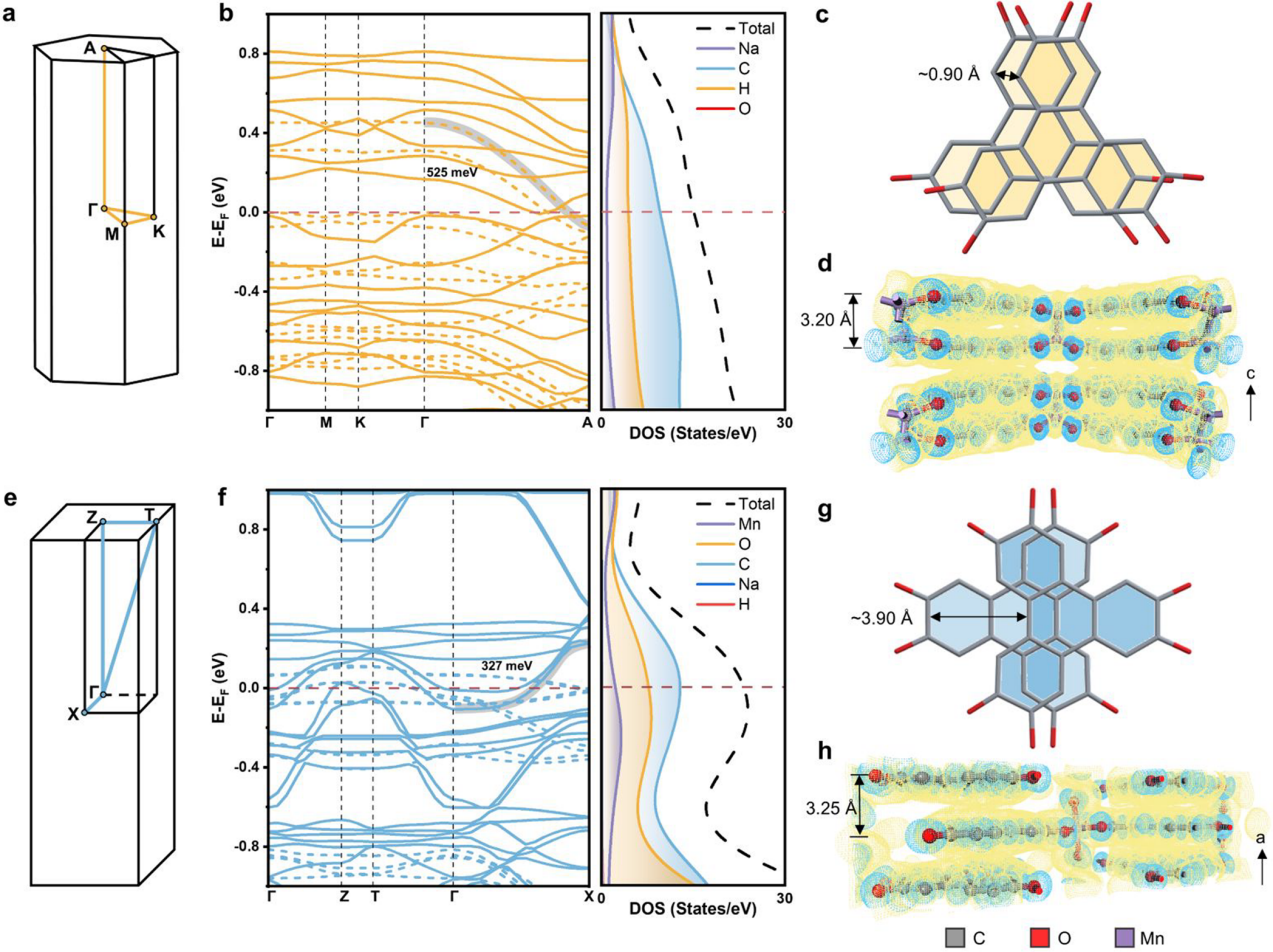
Illustration of Brillouin zone and K-points for (a) Mn-HHTP-**kgm** and (e) Mn-HHTP-**bex-d**. Band structure (solid and dashed lines for spin-up and spin-down electron associated bands, respectively) and PDOS calculated at the DFT-GGA-PBE level of theory using the optimized structures for (b) Mn-HHTP-**kgm** and (f) Mn-HHTP-**bex-d**. Packing models of two adjacent ligands along the π–π stacking in (c) Mn-HHTP-**kgm** and (g) Mn-HHTP-**bex-d**, showing the stacking distortion and spatial shifts. Electron density difference maps of (d) Mn-HHTP-**kgm** and (h) Mn-HHTP-**bex-d** in the out-of-plane direction. The blue and yellow areas represent charge accumulation and depletion, respectively. The electron density isosurface value is 0.02 e/Å^3^. All H atoms are omitted for clarity.

We performed room-temperature electronic conductivity measurements on pelletized samples using the four-point probe method (see Supporting Information for details) to illustrate the influence of topology on electron conduction experimentally. Despite the larger crystallites of Mn-HHTP-**bex-d**, Mn-HHTP-**kgm** demonstrated a 35,000-fold higher electronic conductivity (σ_e_) up to 8.4 × 10^–1^ S cm^–1^ than Mn-HHTP-**bex-d** whose electronic conductivity was determined to be 2.4 × 10^–5^ S cm^–1^ at room temperature (∼298 K) (Figure 4a). Furthermore, temperature-dependent electronic conductivity measurements exhibited typical Arrhenius behavior, with electronic conductivity increasing as temperature increased (Figures S37 and S38). The calculated activation energies for electron conduction were 92 and 363 meV for Mn-HHTP-**kgm** and Mn-HHTP-**bex-d**, respectively (Figure 4b). The aforementioned evidence supports the semiconducting properties of the MOFs or the intergranular hopping between metallic particles. The significantly high electronic conductivity and low activation energy observed in Mn-HHTP-**kgm** can be attributed to the well-aligned triphenylene layers within its structure, which facilitate efficient π–π interactions and interlayer electron delocalization as predicted by DFT calculations. Beyond this study, the values of electronic conductivity and activation energy for Mn-HHTP-**kgm** remain competitive among typical electrically conductive MOFs (Table S4).^19,39,58–63^

**Figure 4:**
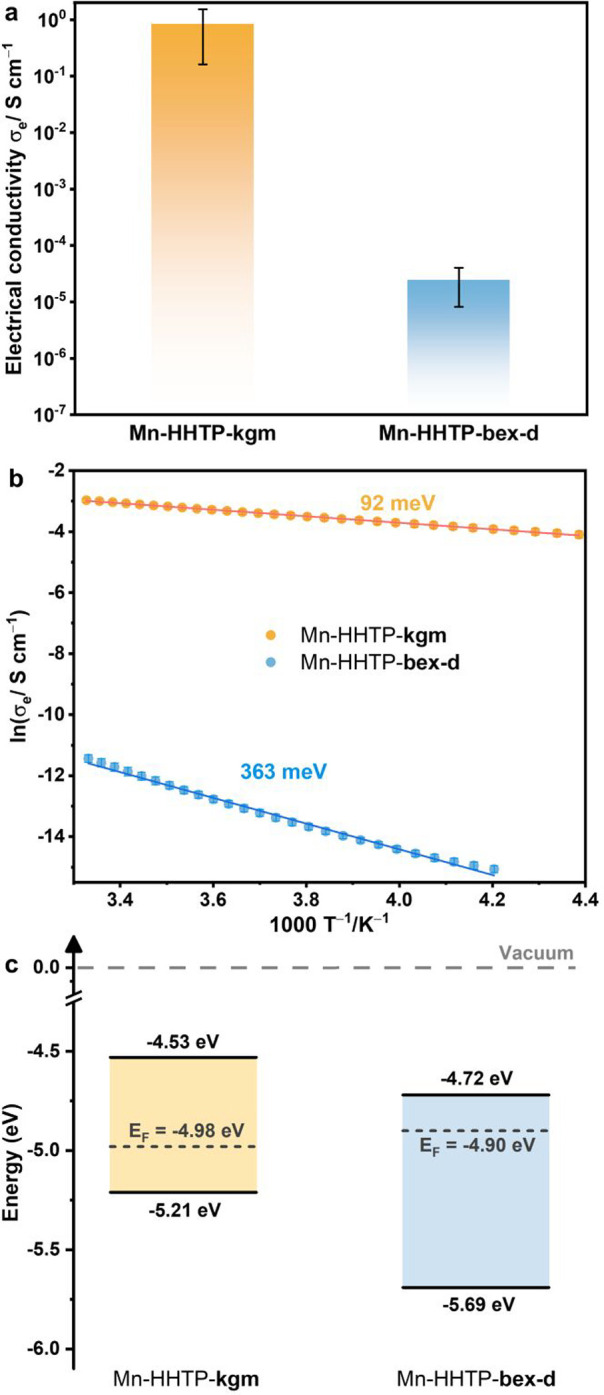
(a) Four-point probe electronic conductivity of Mn-HHTP-**kgm** and Mn-HHTP-**bex-d** at room temperature (∼298 K). (b) Temperature dependence of the electronic conductivities of the pressed pellet of Mn-HHTP-**kgm** and Mn-HHTP-**bex-d** MOFs in Arrhenius coordinates under vacuum. (c) Experimentally determined optical band structures of Mn-HHTP-**kgm** and Mn-HHTP-**bex-d**. The Fermi level (*E*_F_) for each material is indicated.

To further evaluate the conducting nature, optical bandgaps were estimated using Tauc plots derived from ultraviolet–visible–near-infrared (UV–vis–NIR) absorption spectra (Figures S39 and S40). Both MOFs exhibited broad absorption bands extending into the NIR region. The Tauc plots for direct allowed transitions yielded optical bandgaps of 0.68 eV for Mn-HHTP-**kgm** and 0.97 eV for Mn-HHTP-**bex-d**, indicating their semiconducting character. Furthermore, the band structures of both materials were determined using a combination of UV–vis–NIR and ultraviolet photoelectron spectroscopy (UPS) analyses (Figure S41). Similar to other triphenylene-based MOFs with kagome lattice (e.g., Cu_3_(HHTP)_2_, Ni_3_(HITP)_2_), the experimentally determined band structure of Mn-HHTP-**kgm** indicated a p-type semiconducting behavior with the Fermi level (*E*_F_) close to the Valence band. On the other hand, the pseudo **bex-d** topology exhibited a deeper valence band (−5.69 eV) than Mn-HHTP-**kgm** (−5.21 eV), suggesting a tendency to switch from p-type to n-type semiconducting behavior. The Hall effect measurement confirmed the dominant p-type charge carriers in Mn-HHTP-**kgm** with a carrier mobility of ∼0.85 cm^2^ V^–1^ s^–1^ (Figures S42 and S43). These findings demonstrate that framework topology plays a pivotal role in modulating the electronic structure and transport properties of MOFs.

A discrepancy emerges when comparing the pristine, periodic DFT calculations with macroscopic experimental transport data, particularly regarding the metallic versus semiconducting nature of these frameworks. Notably, this mismatch has been documented in both pelletized polycrystalline and single-crystal MOF materials.^64–66^ While DFT predicts metallic or semimetallic band structures for both materials, the experimental temperature-dependent conductivity profiles together with the UV–vis-NIR and UPS spectra demonstrate thermally activated, semiconducting properties. This divergence can be rationalized by a combination of electronic underestimations and extrinsic factors that alter macroscopic transport. On the one hand, DFT calculations can underestimate the electronic band structure, and they are often based on overly idealized models.^65,67^ Extrinsically, factors such as grain boundaries and structural disorders can suppress the macroscopic transport of pelletized polycrystalline samples.^66^ This deviation is particularly pronounced in Mn-HHTP-**bex-d**. While the lower band curvature and diminished dispersion of Mn-HHTP-**bex-d** could qualitatively support the lower electronic conductivity than Mn-HHTP-**kgm**, the experimental value of 10^–5^ S cm^–1^ is too resistive to be consistent with the metallic transport from the DFT calculations. This severe resistive behavior can be ascribed to the highly disruptive grain boundaries and some structural disorders introduced by its limited structural stability, which likely force macroscopic charge transport to proceed via a highly hindered hopping mechanism.

### Proton Conductivity

Examples of mixed-conducting MOFs remain scarce to date with possibilities underexplored. One possible challenge is incorporating proton-conducting functional groups into the conjugated frameworks. The presence of carboxylate moieties and coordinated water molecules within the framework of Mn-HHTP-**bex-d** is proposed to facilitate the proton transport through hydrogen-bonding networks. Hence, we explored the proton conductivity (σ_H_) of the pressed pellets of both Mn-HHTP-**kgm** and Mn-HHTP-**bex-d** via alternating-current (AC) electrochemical impedance spectroscopy (EIS). A two-point probe setup with Nafion membranes as blocking electrodes was employed to exclude the possibility of a Faradaic process (Figure S44). Proton conductivity measurements of both structures were conducted under 98% RH and air, and at various temperatures ranging from 298 to 343 K (Figures 5a,c and S45). Before measurements, the pelletized samples were placed in a chamber at 98% RH and room temperature for 24 h to ensure full hydration and toobtain stable proton conductivity and EIS patterns. The Nyquist plots exhibited an incomplete semicircle at high frequencies and a characteristic tail at low frequencies, fitting well with the equivalent circuit models for proton conduction (Figure S46). Some selected EIS spectra resolved an additional semicircle at intermediate frequencies, which was attributed to grain boundary contributions (Figures S44 and S46c–e). Upon the increase of temperatures, the proton conductivity of Mn-HHTP-**kgm** was observed to increase from 3.6 × 10^–7^ S cm^–1^ (298 K, 98% RH) to 1.4 × 10^–5^ S cm^–1^ (343 K, 98% RH), while Mn-HHTP-**bex-d** showed proton conductivity increasing from 4.5 × 10^–5^ S cm^–1^ (298 K, 98% RH) to 4.7 × 10^–4^ S cm^–1^ (343 K, 98% RH). Accordingly, the least-squares fitting to the Arrhenius equation of proton conductivities demonstrated a linear fit and the activation energies (*E*_a_) were estimated as 0.80 and 0.43 eV for Mn-HHTP-**kgm** and Mn-HHTP-**bex-d**, respectively (Figure 5b). For Mn-HHTP-**kgm**, such a large *E*_a_ (*E*_a_ > 0.4 eV) suggests the vehicular mechanism for proton transport, where diffusion is predominantly through protic species (e.g., H_3_O^+^) as a proton-attached vehicle. For Mn-HHTP-**bex-d**, the magnitude of *E*_a_ value close to the boundary between the Grotthuss (*E*_a_ < 0.4 eV) and vehicular mechanisms (*E*_a_ > 0.4 eV) suggested a mixed-type mechanism. The more than 100-fold increase in proton conductivity at ∼298 K and the lower activation energy of Mn-HHTP-**bex-d** than that of Mn-HHTP-**kgm** confirmed the successful functionalization of electrically conductive MOFs via coordinating metal nodes with functional groups. Since the proton conductivity of Mn-HHTP-**bex-d** approaches the magnitude of its electronic conductivity, previous electronic conductivity measurements were re-evaluated to rule out the protonic interference. Proton conduction in Mn-HHTP-**bex-d** is strictly humidity-dependent (Figure S47). At low humidity (<50% RH), the proton conductivity can drop by approximately 1–2 orders of magnitude compared to the value at 98% RH, suggesting that the electronic conductivity, rather than proton conductivity, dominates macroscopic charge transport under ambient conditions, and the corresponding effects of proton conduction on charge transport are considered minimal. Furthermore, the resistance calculated from the steady-state current of the chronoamperometry test matched well with the values derived from *I*-*V* determinations (Figure S48), validating the accuracy of the electronic conductivity measurements under ambient conditions.

**Figure 5:**
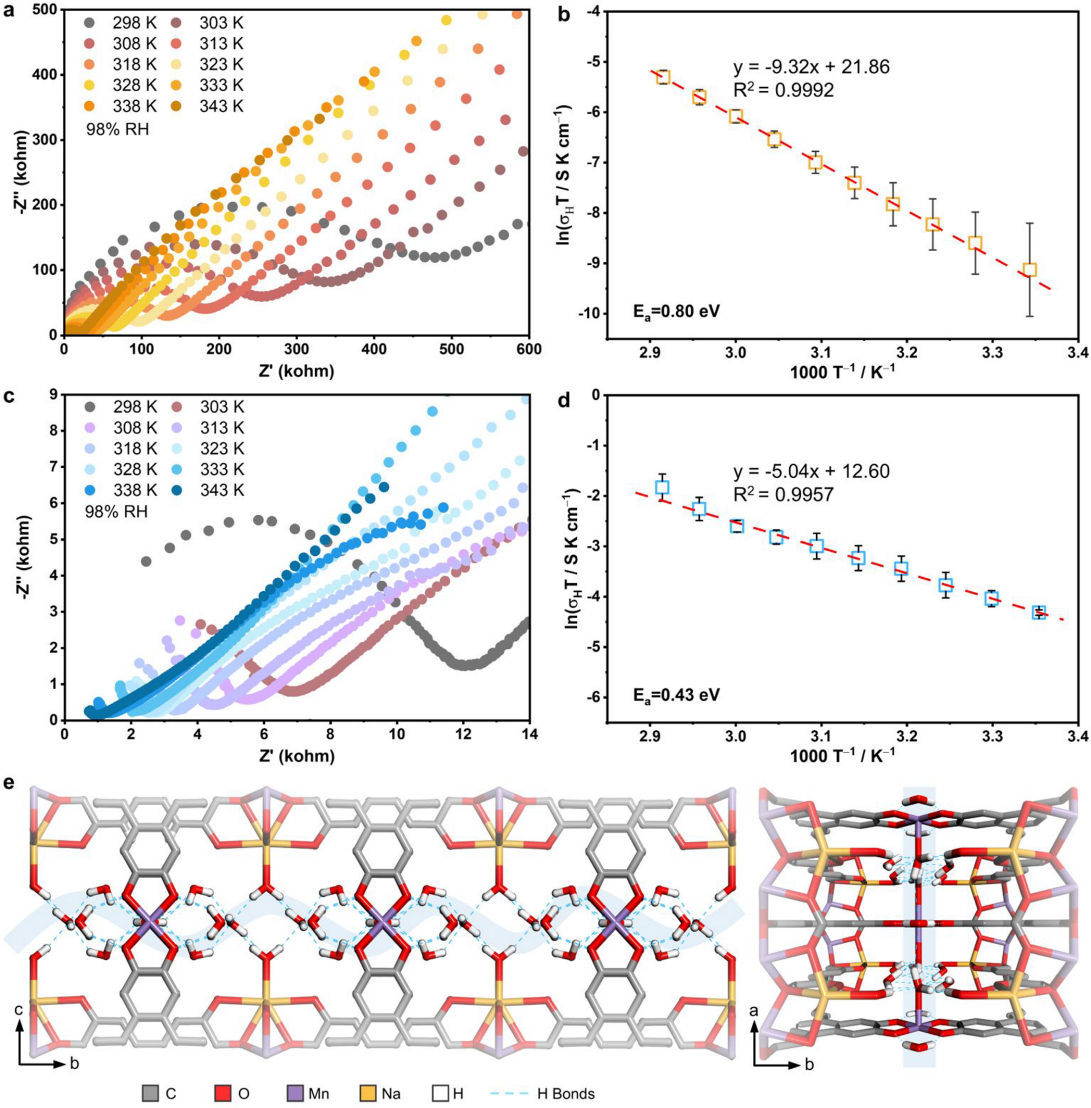
(a) Nyquist plots measured with 98% RH at different temperatures and (b) Corresponding Arrhenius plots for pelletized Mn-HHTP-**kgm**. *E*_a_ stands for the calculated activation energy. (c) Nyquist plots measured with 98% RH at different temperatures and (d) Corresponding Arrhenius plots for pelletized Mn-HHTP-**bex-d**. Open squares are experimental data, and the dashed line is the fitting result. (e) Representative of SCXRD-resolved organizations of water molecules and hydrogen-bond networks in the channels of Mn-HHTP-**bex-d** as viewed along the *a* axis and *c* axis. H atoms on HHTP ligands and acetate are omitted for clarity.

Proton transport in the polycrystalline pellets can stem from both intrinsic structural pathways within the porous materials and grain boundaries between crystallites. To gain a clear understanding and valuable insights into proton transport in Mn-HHTP-**bex-d**, quantitative deconvolution of the selected EIS spectra was performed to isolate these components (Figure S46c–e), revealing that grain resistances were consistently lower than grain boundary resistances. This highlights the dominant role of the intrinsic framework structure of Mn-HHTP-**bex-d** in facilitating proton transport. Based on the crystallographic structure derived from single-crystal analysis of Mn-HHTP-**bex-d**, water molecules occupy the pores and channels upon hydration with an additional uptake of up to 2 water molecules per structural formula unit, corresponding to Mn_3_Na_2_(HHTP)_2_(CH_3_COO)_2_(H_2_O)_4_·2H_2_O. These adsorbed water molecules, together with the coordinated H_2_O, acetate, and HHTP linkers, form an extended O–H···O hydrogen-bond network for proton transport (Figures 5e and S49). It should be noted that evaluating transport properties on a single-crystal device would more effectively exclude the effect of grain boundaries and further emphasize the intrinsic structure–property relationship in future studies. After exposure to the hot and humid environment, the crystallinity of Mn-HHTP-**kgm** decreased significantly, whereas Mn-HHTP-**bex-d** retained structural integrity to some extent (Figure S50). The moisture sensitivity observed in the **kgm** topology can be ascribed to the rapid water–ligand exchange at the dimeric paddle-wheel Mn­(II) nodes.^68,69^

Layered triphenylene-based conjugated MOFs are well-known for their electronic conductivity, but are typically poor in proton conductivity. For example, Park et al. reported that Cu_3_(HHTP)_2_ did not exhibit measurable room-temperature proton conductivity even at 95% RH, while the proton conductivity of Zn-HHTP-H_2_O remained limited to the order of 10^–6^ S cm^–1^ despite the presence of axial water molecules. Consequently, the sluggish proton transport in Mn-HHTP-**kgm** with a conventional structure is consistent with these benchmarks, yielding a proton conductivity (3.6 × 10^–7^ S cm^–1^ at 298 K, 98% RH) approximately 1,000,000-fold lower than its electronic counterpart. The stark disparity between electronic and protonic conductivities restricts the ambipolar conductivity (defined as $\frac{\sigma_{e}\sigma_{H}}{\sigma_{e}+\sigma_{H}}$ ) of Mn-HHTP-**kgm** to only the order of 10^–7^ S cm^–1^. Instead, Mn-HHTP with a pseudo **bex-d** topology achieved a more balanced profile for a dual proton–electron conductor to achieve high ambipolar conductivity of ∼1.6 × 10^–5^ S cm^–1^, outperforming other reported MOFs with mixed conductivity (Figure 6 and details in Table S4).^1–3,7,12,24–26,29,70^ An add-in highlight over other mixed-conducting MOFs is that the proton-conducting pathways in Mn-HHTP-**bex-d** were clearly revealed by crystallographic analysis.

**Figure 6:**
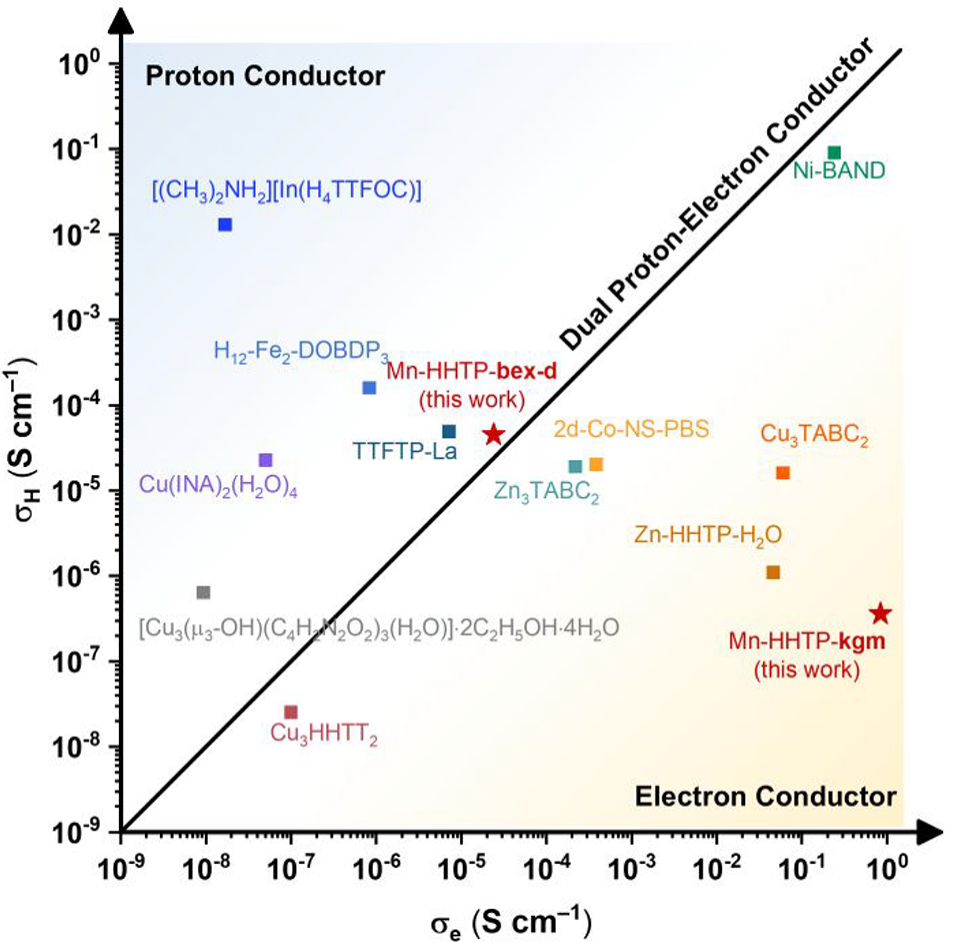
Comparison of the mixed proton–electron conduction (σ_e_: electronic conductivity; σ_H_: proton conductivity) of Mn-HHTP-**kgm** and Mn-HHTP-**bex-d** with those of reported MOFs. The dual proton–electron conductor requires a balanced conductivity (σ_e_/σ_H_ = ∼1).

## Conclusions

In summary, we demonstrated a strategy for tuning the electronic and protonic conductivities of MOFs through topological control. By modulating synthetic conditions, we isolated two distinct layered conjugated frameworks constructed from identical Mn­(II) nodes and triphenylene-derived linkers (HHTP) in a 3:2 ratio. While Mn-HHTP-**kgm** resembles traditional triphenylene-based 2D MOFs with serrated AA′ stacking, featuring kagome lattice and hexagonal pores, the successfully grown single crystal of **Mn-HHTP-bex-d** reveals a unique, unprecedented **pseudo bex-d topology** with a 2-nodal net. Our findings establish a clear structure–property relationship, indicating the effectiveness of topological control in tuning the charge transport properties within MOFs. Specifically, Mn-HHTP-**kgm** with well-aligned, serrated-parallel π–π stacking and small interlayer spacing achieved an excellent room-temperature electronic conductivity of 8.4 × 10^–1^ S cm^–1^, representing an improvement of 4 orders of magnitude compared to Mn-HHTP-**bex-d** (2.4 × 10^–5^ S cm^–1^). In contrast, due to the coordination of Mn­(II) nodes with water molecules and acetate ions, Mn-HHTP-**bex-d** incorporates abundant hydrophilic sites and hydrogen bond networks revealed by crystallographic analysis, resulting in a nearly 100-fold increase in proton conductivity compared to Mn-HHTP-**kgm** (σ_H_ = 3.6 × 10^–7^ S cm^–1^ for Mn-HHTP-**kgm**; σ_H_ = 4.5 × 10^–5^ S cm^–1^ for Mn-HHTP-**bex-d** at 298 K, 98% RH). Unlike the starkly divergent conductivities observed in **kgm** topology, the balanced electronic and protonic transport in Mn-HHTP-**bex-d** evidences the successful integration of dual-conduction pathways within a single-phase MOF. This study elucidates the decisive role of structural topology in dual electron–proton transport performance, providing new insights for designing multifunctional MOF materials applicable to ambipolar field-effect transistors, bioelectronic interfaces, and various energy devices. Furthermore, the newly illustrated topology enriches the structural family of reticular chemistry, providing new paradigms for constructing complex framework materials.

## Supplementary Material



## Notes

Experimental procedures, characterization data, DFT computation details, and crystallographic data (PDF)
