# Supplementary material for: Topological Control of Dual Protonic–Electronic Conduction in Metal–Organic Frameworks
Source: J Am Chem Soc. 2026 Jul 2;148(27):28742–53. doi: 10.1021/jacs.6c05964 (PMC13383715; doi:10.1021/jacs.6c05964)
Supplement: Supplementary file 1 [file ja6c05964_si_001.pdf]

Supporting Information

Topological Control of Dual Protonic–Electronic Conduction in Metal–Organic Frameworks

Huilin Qing,[a,†] Priyanshu Chandra,[b,†] Joseph Y. M. Chan,[b] Richard J. Staples,[c] Tai-De Li,[d,e] Weiyang Li,[a,*] and Katherine A. Mirica[b,*]

Author Affiliations:

[a] Thayer School of Engineering, Dartmouth College, Hanover, New Hampshire 03755, United States

[b] Department of Chemistry, Dartmouth College, Hanover, New Hampshire 03755, United States

[c] Department of Chemistry, Michigan State University, East Lansing, Michigan 48824, United States

[d] Nanoscience Initiative, Advanced Science Research Center, CUNY Graduate Center, City University of New York, New York 10031, United States

[e] Department of Physics, City College of New York, City University of New York, New York 10031, United States

[†] These authors contribute equally.

*weiyang.li@dartmouth.edu

*katherine.a.mirica@dartmouth.edu

Table of Contents

1. Materials and Synthesis 3
1.1 Materials 3
1.2 Optimized synthesis of Mn-HHTP-kgm 3
1.3 Synthesis and characterization of Mn-HHTP-bex-d MOF 6
2. Crystal Structure and Materials Characterizations 7
2.1 General characterization method 7
2.2 Simulated Structure of Mn-HHTP-kgm 8
2.3 Crystal structure of Mn-HHTP-bex-d 14
2.4 SEM images of Mn-HHTP-kgm and Mn-HHTP-bex-d 21
2.5 TEM image of Mn-HHTP-kgm 23
2.6 Brunauer–Emmett–Teller (BET) Analysis 24
2.7 EPR 25
2.8 XPS experiments 26
2.9 FT-IR 28
2.10 Structural stability of Mn-HHTP-kgm and Mn-HHTP-bex-d 29
3. Computational Study for structure simulation and electronic band structure 31
4. Electronic conductivity and electronic structure 35
4.1 Room-temperature electronic conductivity measurements 35
4.2 Variable temperature electronic conductivity data 36
4.3 Room-temperature electrical conductivity comparison 39
4.4 Electronic Structure 41
4.5 Hall measurements 43
5. Proton conductivity measurements 45
6. References 52

1. Materials and Synthesis

1.1 Materials

Manganese (II) acetate tetrahydrate, manganese(II) nitrate tetrahydrate, manganese (II) acetate and sodium acetate were purchased from Thermo Fisher Scientific. 2,3,6,7,10,11-hexahydroxy triphenylene (HHTP) was purchased from TCI chemicals. Ethanol and acetone were purchased from BDH Chemicals. N,N-Dimethylformamide (DMF) was purchased from Sigma-Aldrich. Deionized water was used for all experiments.

1.2 Optimized synthesis of Mn-HHTP-kgm

$Mn(OAc)_2·4H_2O$ (150 mg, 0.6 mmol) is dissolved into 80 mL water in a 100 mL pressure vial. Then, HHTP (80 mg, 0.24 mmol) is suspended in the above solution. The pressure vial was closely capped, and the resulting mixture was kept at 80 °C for 20 hrs. The black solids are collected through centrifugation and thoroughly washed with ethanol for four times. The solids are dried in vacuum oven of 70 °C. The as-synthesized materials were activated at 100 °C under vacuum for 24 hrs in prior to N_2 sorption measurement. Some synthesis optimizations are provided below.

Table S1. Synthesis optimization table for $Mn_3(HHTP)_2$ (Mn-HHTP-**kgm**) MOF.

No.	Usage of HHTP	Conc. Of $Mn(OAc)_2·4H_2O$	Additives	Solvents	Notes
1	3 mM	7.5 mM	-	H_2O	Uncapped, excess O_2
2	3 mM	7.5 mM	7.5 mM $Mn(NO_3)_2$	H_2O	-
3	3 mM	15 mM	-	H_2O	-
4	3 mM	7.5 mM	-	DMF	-
5	3 mM	7.5 mM	-	DMF:H_2O (1:1)	-
6	3 mM	7.5 mM	-	DMF:H_2O (1:4)	-
7	3 mM	7.5 mM	-	Ethanol	-
8	6 mM	15 mM	-	H_2O	-
9	3 mM	7.5 mM	-	H_2O	N_2
10	3 mM	7.5 mM	3 mM CH_3COONa	H_2O	-
11	10 mM	-	50 mM $Mn(OAc)_2$ and 200 mM CH_3COONa	DMF	N_2
12	10 mM	-	50 mM $Mn(OAc)_2$	DMF	N_2
13	3 mM	7.5 mM	-	H_2O	Optimized condition

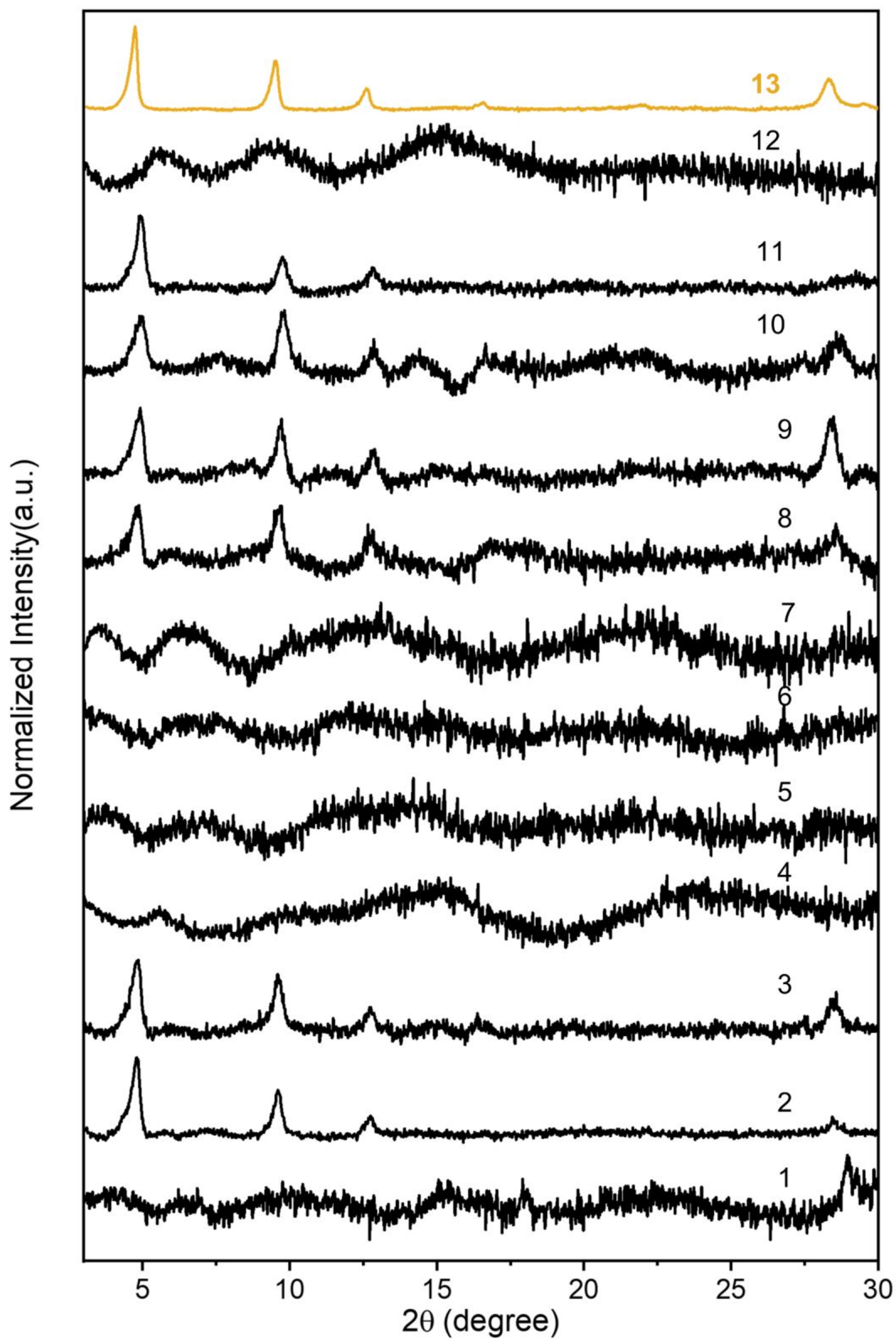

Figure S1. PXRD spectra of product obtained from synthetic optimization of $Mn_3(HHTP)_2$ MOF (Mn-HHTP-**kgm**).

1.3 Synthesis and characterization of Mn-HHTP-bex-d MOF

Growing single crystals of Mn-HHTP-**bex-d** MOF: HHTP (160 mg, 0.5 mmol) and $Mn(NO_3)_2.4H_2O$ (310 mg, 1.25 mmol) are charged into a 250 mL RBF to which 50 mL DI water is added. The resulting mixture is bubbled with N_2 and is stirred for 2 hr at room temperature. CH_3COONa (2.46 g, 30 mmol) in 7mL DI water is then introduced to the mixture under N_2 atmosphere, and the reaction is further stirred with N_2 bubbling for 1 hr. Finally, stirring and N_2 bubbling are turned off, and the reaction mixture is heated at 80 °C for 24 hr. After completion of the reaction, needle-shaped, dark-blue crystals are obtained by filtration and washed with water and acetone. The structure of the crystal is solved by single-crystal X-ray diffraction (SCXRD) measurement. SCXRD measurement revealed that the structure of the MOF is completely different from that of the earlier synthesized MOF. The solved crystal structure of Mn-HHTP-**bex-d** has been uploaded to CCDC dataset with a deposition number of 2534551.

2. Crystal Structure and Materials Characterizations

2.1 General characterization method

Powder X-ray diffraction (pXRD) was performed with a Rigaku sixth generation MiniFlex X-ray diffractometer with a 600 W (40 kV, 15 mA) Cu-Kα radiation (λ = 1.54 Å) radiation source. Scanning Electron Microscopy (SEM) and Energy Dispersive X-ray Spectroscopy (EDS) were performed using a Thermo Scientific™ Helios™ 5 CX DualBeam with an Ultim Max EDS detector. Transmission electron microscopy (TEM) was carried out at an FEI Talos™ f200i S/TEM instrument under 200 kV beam voltage. Nitrogen adsorption/desorption measurements at 77 K were performed on a Micromeritics 3Flex to obtain pore properties such as the specific surface area, total pore volume, and pore size distribution. Thermal gravimetric analysis (TGA) was performed using a TA Instruments TGA Q150 with a 5 °C/min ramp from room temperature to the target temperature under N_2 or Air. Fourier transform-infrared spectroscopy (FT-IR) measurements for powder materials were performed on a Nicolet iS50 FT-IR spectrometer with deuterated doped triglycine sulfate (DTGS) detector. X-ray photoelectron spectroscopy (XPS) experiments were conducted on Kratos Analytical AXIS Supra X-ray Photoelectron Spectrometer under ultrahigh vacuum (base pressure 1×10^{-7} Torr). The measurement chamber was equipped with a monochromatic Al (Kα) X-ray source. A beam diameter of 200 μm was used for wide-scan survey and high-resolution spectra. EPR spectra were collected on a Bruker BioSpin GmbH spectrometer equipped with a standard mode cavity. For Ultraviolet-Visible-Near Infrared (UV-vis-NIR) spectra of samples, the MOF in H_2O was dropcasted on the quartz slides. UV-vis-NIR between 350 and 2500 nm was collected on a JASCO V-570 spectrophotometer at a scan rate of 200 nm min^{-1} under ambient conditions. A blank quartz slide baseline and a zero-background correction were conducted prior to the sample measurement. The ultra-violet photoelectron spectroscopy (UPS) measurements were conducted using ultra-violet UV light option of PHI VersaProbe II X-ray photoelectron spectroscopy. For the UPS measurement, standard gold was used as the control, and each sample was measured at three bias voltages: -5, -10, and -15 V to ensure reliability. Micro electron diffraction (MicroED) analysis was conducted on a Rigaku XtaLAB Synergy-ED diffractometer.

2.2 Simulated Structure of Mn-HHTP-kgm

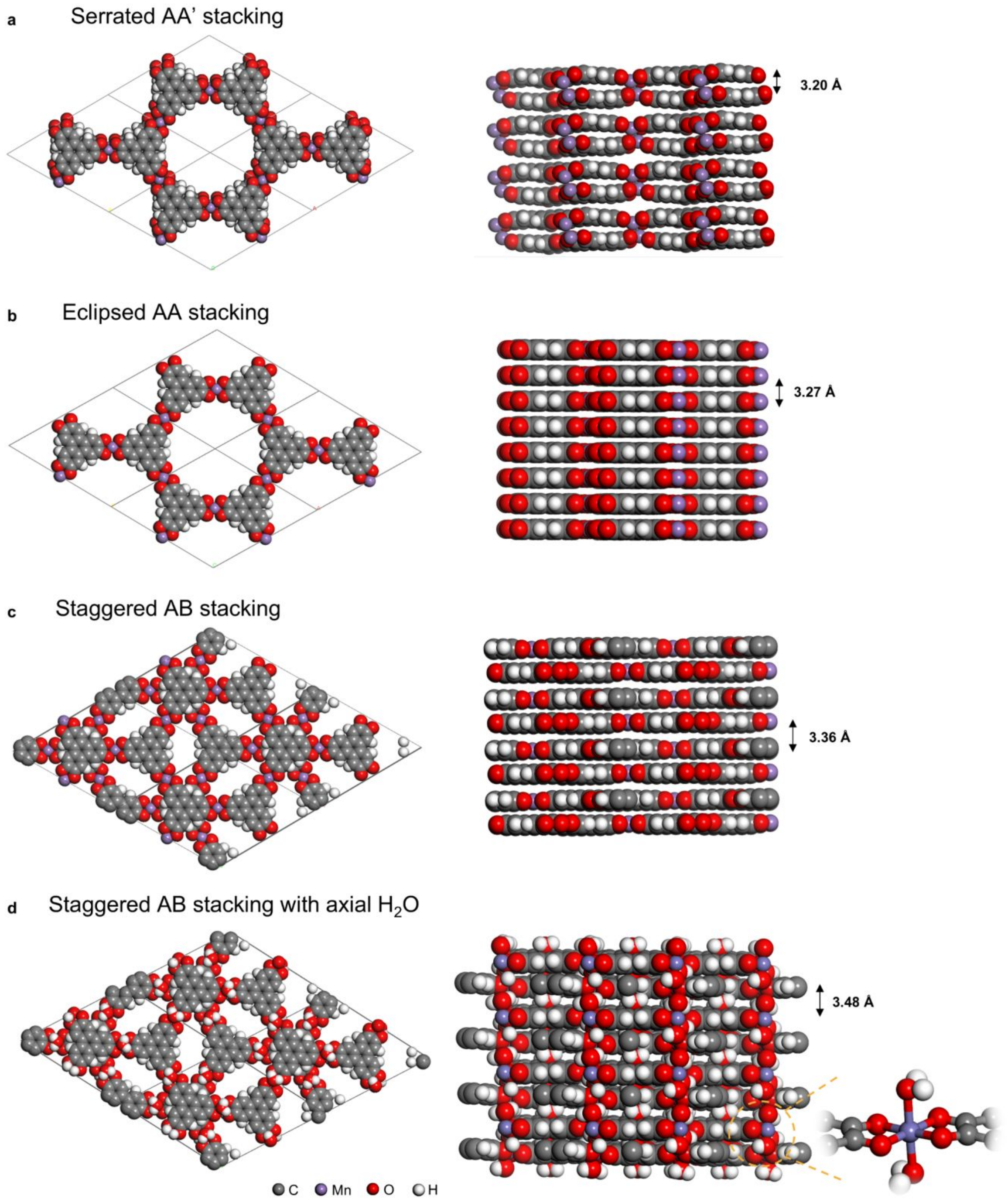

Figure S2. Comparison between different simulated structures of Mn-HHTP-**kgm**. In-plane and out-of-plane views of a) serrated AA' stacking, b) eclipsed AA stacking, c) staggered AB stacking, and d) staggered AB stacking with axial H_2O (axial H_2O is added to each Mn(II) center). Interlayer distances are marked in different structures. A van der Waals correction was consistently applied for the geometry optimization.

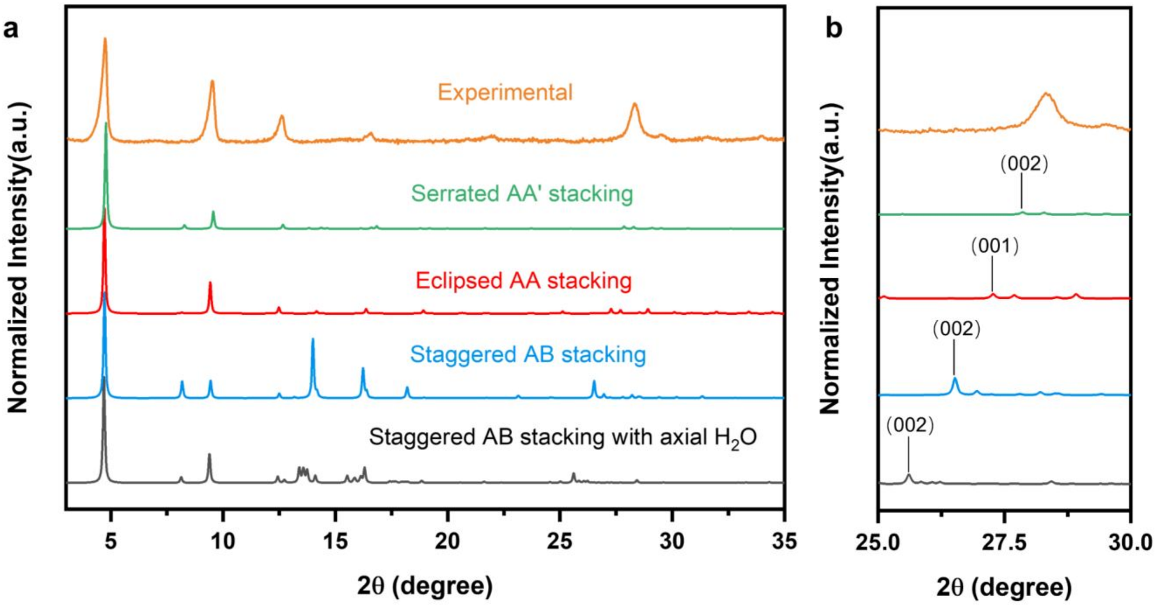

Figure S3. a) Experimental PXRD and simulated PXRD based on different simulated crystal structures (i.e, serrated AA' stacking, eclipsed AA stacking, staggered AB stacking, and staggered AB stacking with axial H_2O). The simulated PXRD pattern of serrated AA' stacking best fit the experimental results. b) Zoom-in patterns in the range of 25.0−30.0°.

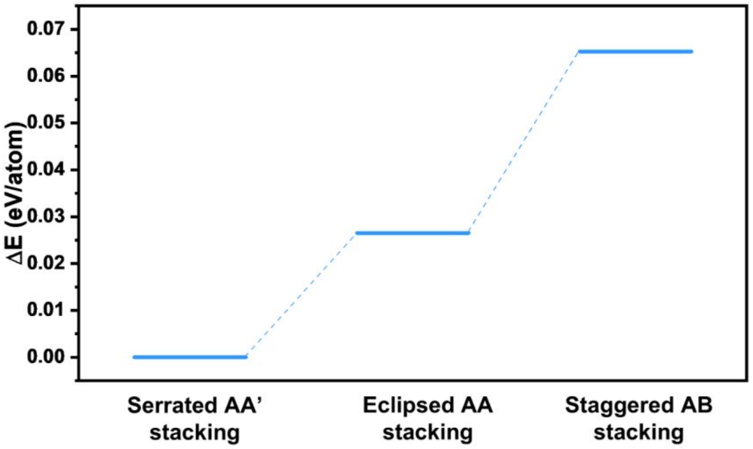

Figure S4. Relative DFT energies (ΔE, per atom in the cell) referenced to serrated AA' stacking.

Justification for the best-modeled structure:

Based on the modeled structures, the staggered AB stacking configuration produces a PXRD pattern that clearly deviates from the experimental results (**Figure S3**). When considering only the 3−20° 2θ range, however, several stacking models (*i.e.*, serrated AA' stacking, eclipsed AA stacking, and staggered AB stacking with axial H_2O) could appear to generate reasonably similar diffraction patterns. However, these candidate models differ significantly in their predicted interlayer spacings. Notably, the experimental PXRD pattern exhibits a distinct reflection at a high angle (~28.3°), which is commonly associated with interlayer spacing in 2D layered MOFs. This reflection is consistently observed across repeated PXRD measurements, and such an angle is noticeably higher than the ~27.3° peak corresponding to a 3.3 Å interlayer spacing in reported 2D $Cu_3(HHTP)_2$ frameworks. The shift to a higher 2θ value indicates a reduced interlayer distance in Mn-HHTP-**kgm**. Among the modeled structures, only the serrated AA' stacking model yields the smallest interlayer spacing (~3.2 Å), which agrees best with the experimental PXRD pattern. In contrast, the other stacking models (3.27, 3.36, and 3.48 Å) would be expected to produce characteristic reflections for 2D layered MOFs at lower 2θ values (<27.3°). The consistent absence of such low-angle reflections in the experimental data supports the serrated AA' stacking model as the best-fit structural configuration. The relative DFT energies in **Figure S4** also indicates the serrated AA' stacking model is the most stable among serrated AA', eclipsed AA and staggered AB stacking models.

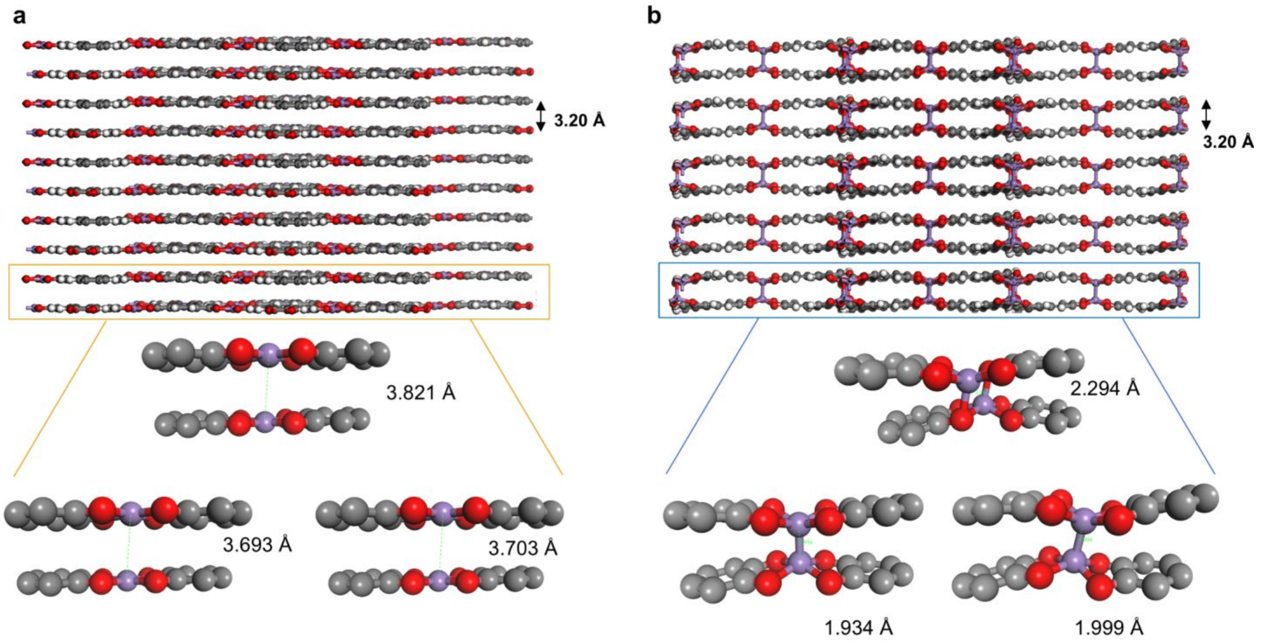

Figure S5. Details about the coordination environment of Mn nodes in the Mn-HHTP-**kgm**: (a) optimized serrated AA' stacking without van der Waals correction and (b) optimized serrated AA' stacking with van der Waals correction. The Mn···Mn distances are indicated in the figure. Some H atoms are omitted for clarity. In (a), Mn···Mn distances are different from the interlayer spacing due to the positional offsets on the xy-plane in the serrated stacking pattern. With a van der Waals correction added for geometry optimization, the interlayer spacing remains unchanged, whereas the coordination geometry of Mn changes from square planar to distorted square pyramid.

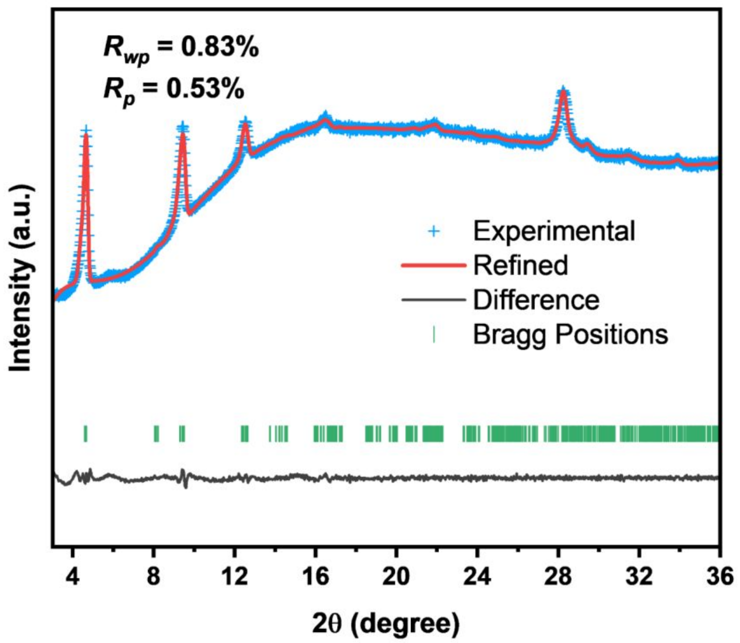

Figure S6. Pawley refinement result of experimental PXRD pattern based on the structure of serrated AA' stacking. The experimental PXRD pattern is used and shown without background subtraction.

Table S2. Lattice parameters of Mn-HHTP-**bex-d** with serrated AA' stacking before and after Pawley refinement.

	**Initial Structure**	**Pawley Refined Structure**
**a**	21.34 Å	21.21 Å
**b**	21.35 Å	21.64 Å
**c**	6.40 Å	6.39 Å
**α**	90.0°	89.6°
**β**	90.0°	89.9°
**γ**	120.0°	119.9°

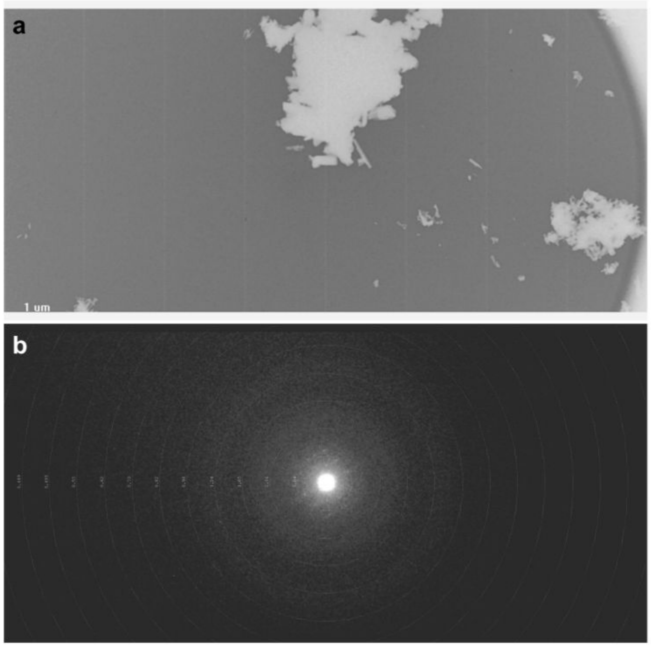

Figure S7. Preliminary results of micro electron diffraction (MicroED) characterization of Mn-HHTP-**kgm**. (a) TEM image of the Mn-HHTP-**kgm** crystals. (b) A preliminary image of the diffraction pattern of Mn-HHTP-**kgm** from a MicroED trial. Despite some diffraction near the center of the detector, the quality of the diffraction data is not sufficient for crystal structure solution.

2.3 Crystal structure of Mn-HHTP-bex-d

Experimental details of single crystal XRD: A brown needle-shaped-shaped crystal with dimensions 0.21×0.07×0.04 mm^3 was picked directly from mother liquor without any further modification. The single crystal was mounted on a nylon loop with paratone oil on a XtaLAB Synergy, Dualflex, HyPix diffractometer. The crystal was kept at a steady T = 100.01(11) K during data collection. The structure was solved with the ShelXT 2018/2 (Sheldrick, 2018) solution program using dual methods and by using Olex2 as the graphical interface. The model was refined with ShelXL 2018/3 (Sheldrick, 2015) using full matrix least squares minimization on F^2.

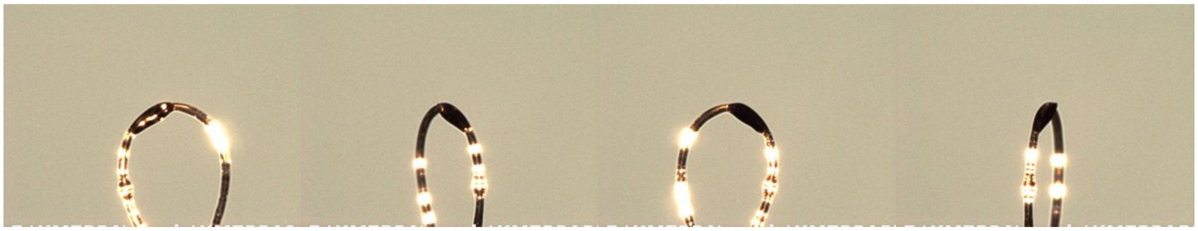

Figure S8. Selected single crystal picture.

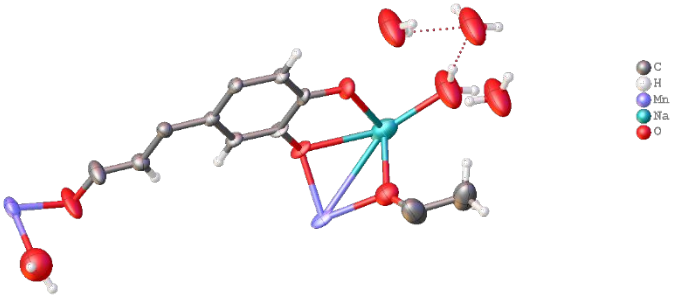

Figure S9. ORTEP illustration of Mn-HHTP-**bex-d** structure.

Table S3. Crystal data and details of Mn-HHTP-**bex-d**.

**Compound**	**Mn-HHTP-bex-d**
Formula	$C_{40}H_{30}Mn_3Na_2O_{22}$
$D_{calc.}$/ g cm^{-3}	1.606
m/mm^{-1}	7.784
Formula Weight	1073.44
Colour	brown
Shape	needle-shaped
Size/mm^3	0.21×0.07×0.04
T/K	100.01(11)
Crystal System	orthorhombic
Space Group	*Cmmm*
a/Å	6.4437(2)
b/Å	27.9526(13)
c/Å	12.3225(4)
a/°	90
b/°	90
g/°	90
V/Å^3	2219.51(14)
Z	2
Z'	0.125
Wavelength/Å	1.54178
Radiation type	Cu K_a
Q_{min}/°	3.162
Q_{max}/°	80.161
Measured Refl's.	12233
Indep't Refl's	1360
Refl's I≥2 *s*(I)	1226
R_{int}	0.0696
Parameters	113
Restraints	0
Largest Peak	1.278
Deepest Hole	-0.770
GooF	1.196
wR_2 (all data)	0.2869
wR_2	0.2805
R_1 (all data)	0.1087
R_1	0.1018

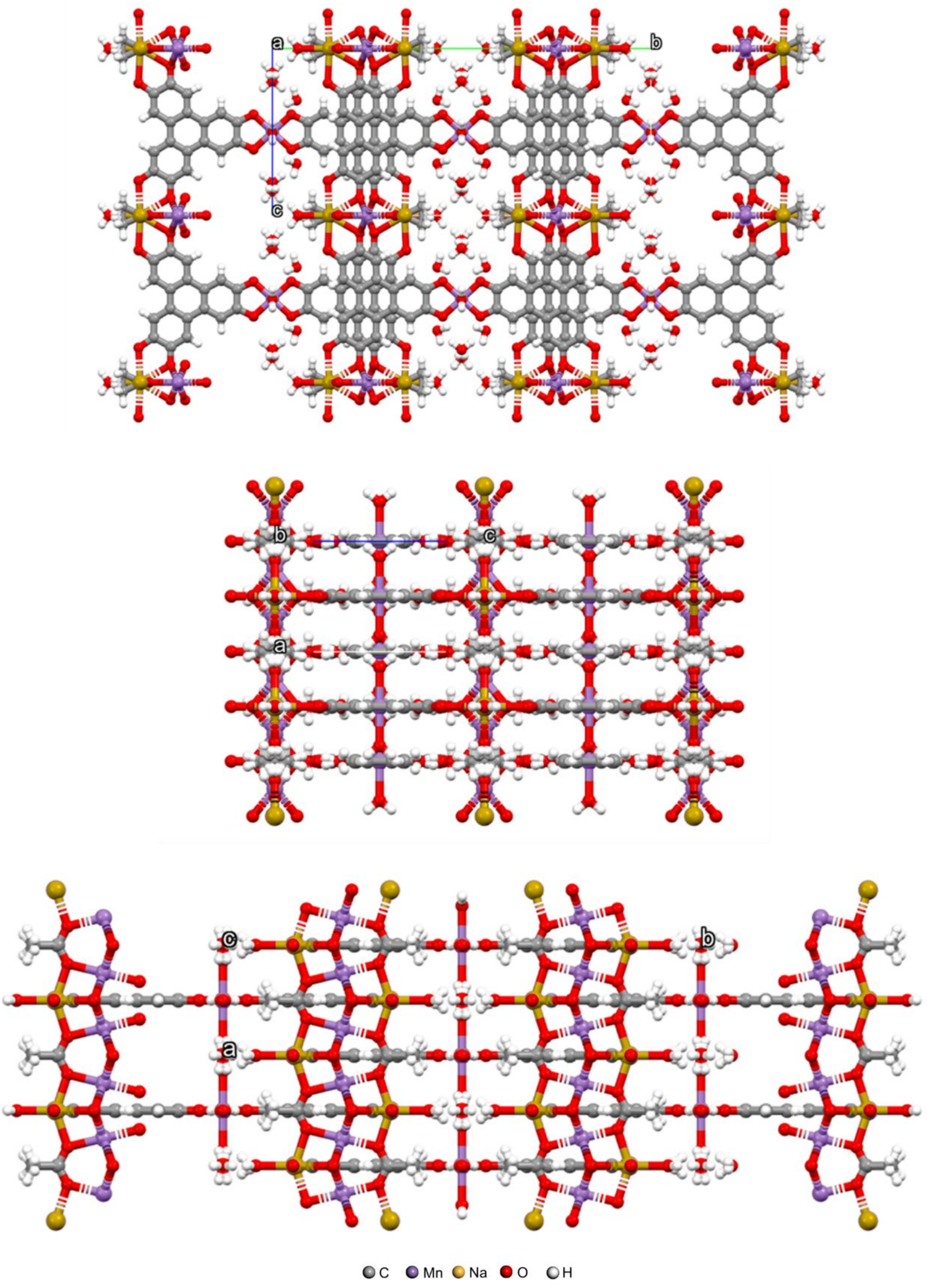

Figure S10. Structures of Mn-HHTP-**bex-d** along a (top), b (middle), and c-axis (bottom).

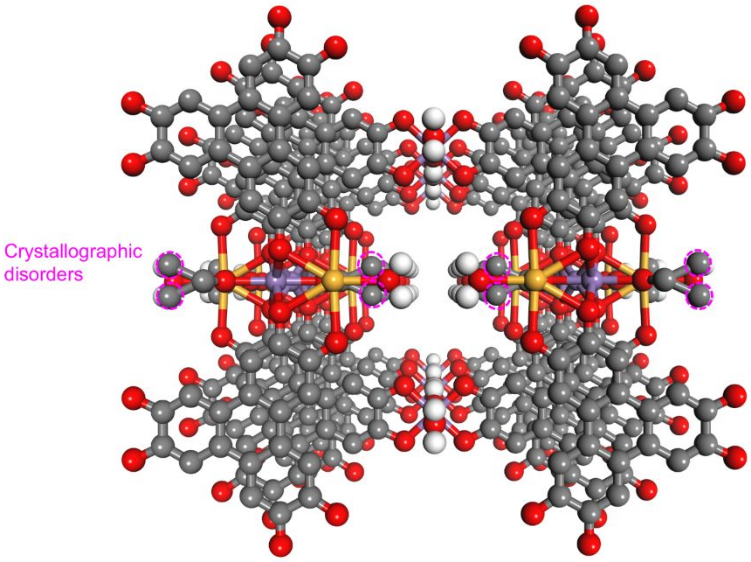

Figure S11. Illustration of crystallographic disorders of methyl groups in acetate.

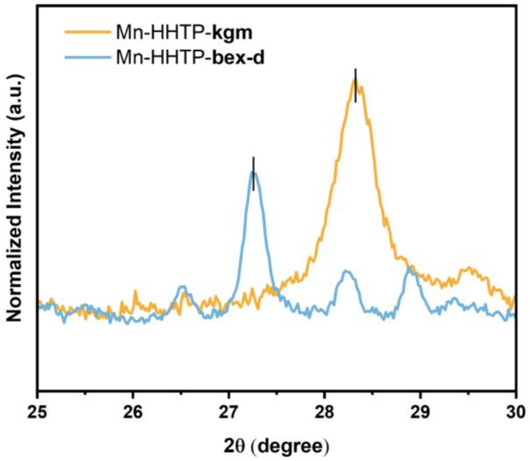

Figure S12. Detailed comparison of interlayer stacking distance between Mn-HHTP-**kgm** and Mn-HHTP-**bex-d**. The peak indicating stacking distance in Mn-HHTP-**bex-d** is located at a smaller degree compared to that in Mn-HHTP-kgm, suggesting a larger stacking distance in **bex-d** topology.

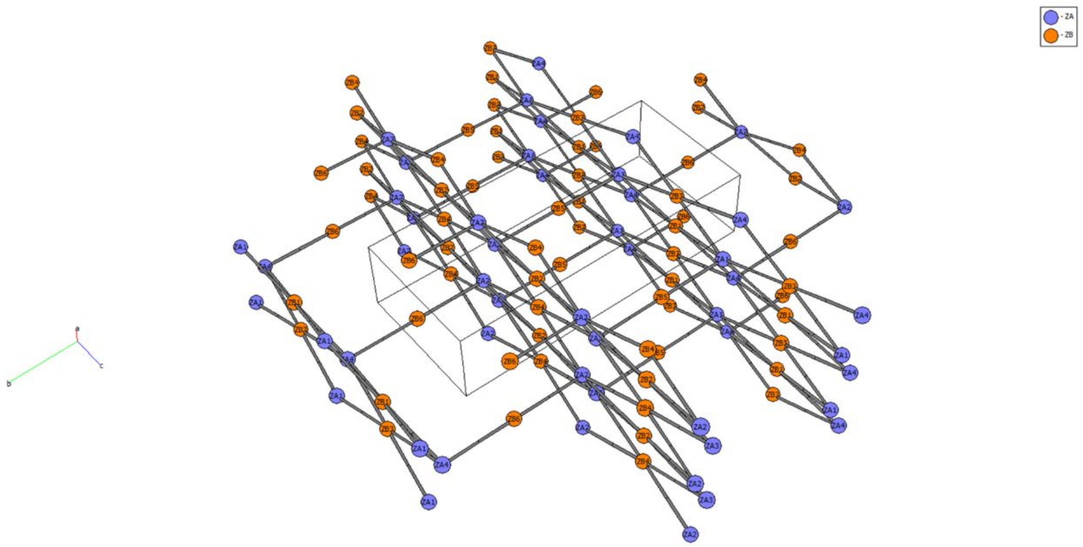

Figure S13. Simplified structure of Mn-HHTP-**bex-d** for topology analysis.

Both Mn(II) and HHTP as central groups

```
Point symbol for net: {4^4.6^2.8^4}2{4^4.6^2}2{8}
Point symbol for net with loops: {4^4.6^2}2{4^4.6^6}2{6}
2,4,5-c net with stoichiometry (2-c)(4-c)2(5-c)2; 3-nodal net

New topology, please, contact the authors (17974 types in 4 databases)
```

Only Mn(II) as central group

```
Point symbol for net: {3^12.4^8.5^8}{3^24.4^34.5^8}2
8,12-c net with stoichiometry (8-c)(12-c)2; 2-nodal net

New topology, please, contact the authors (17974 types in 4 databases)
```

Figure S14. Topological analysis of Mn-HHTP-**bex-d** using ToposPro.These results indicate a new topology that has not been established.

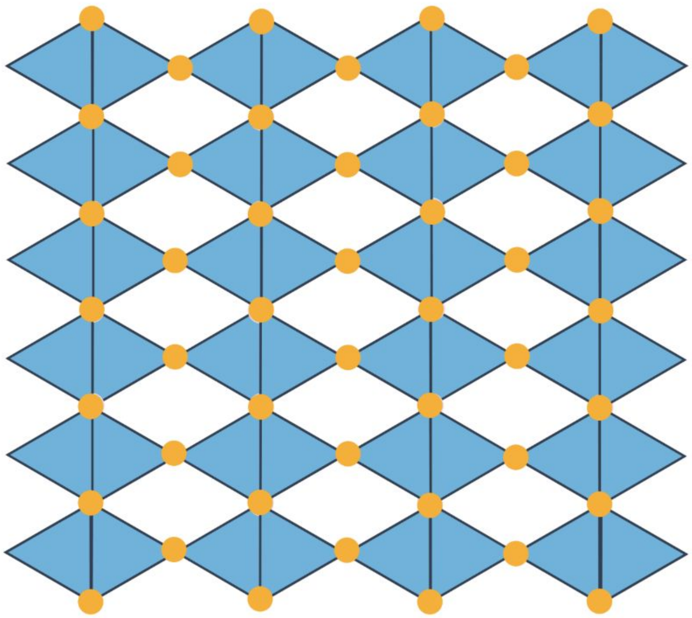

Figure S15. Schematic illustration of typical **bex-d** topology according to Reticular Chemistry Structure Resource (RCSR) website. Typical **bex-d** topology arises from the arrangement of monolayer, while the pseudo **bex-d** topology results from the adjacent bilayers. In 2D bex-d networks, the topology consists of 4- and 6-connected nets, whereas extension into a pseudo-2D bex-d topology results in higher connectivities of 8 and 12, as demonstrated by topological analysis with Mn as central groups.

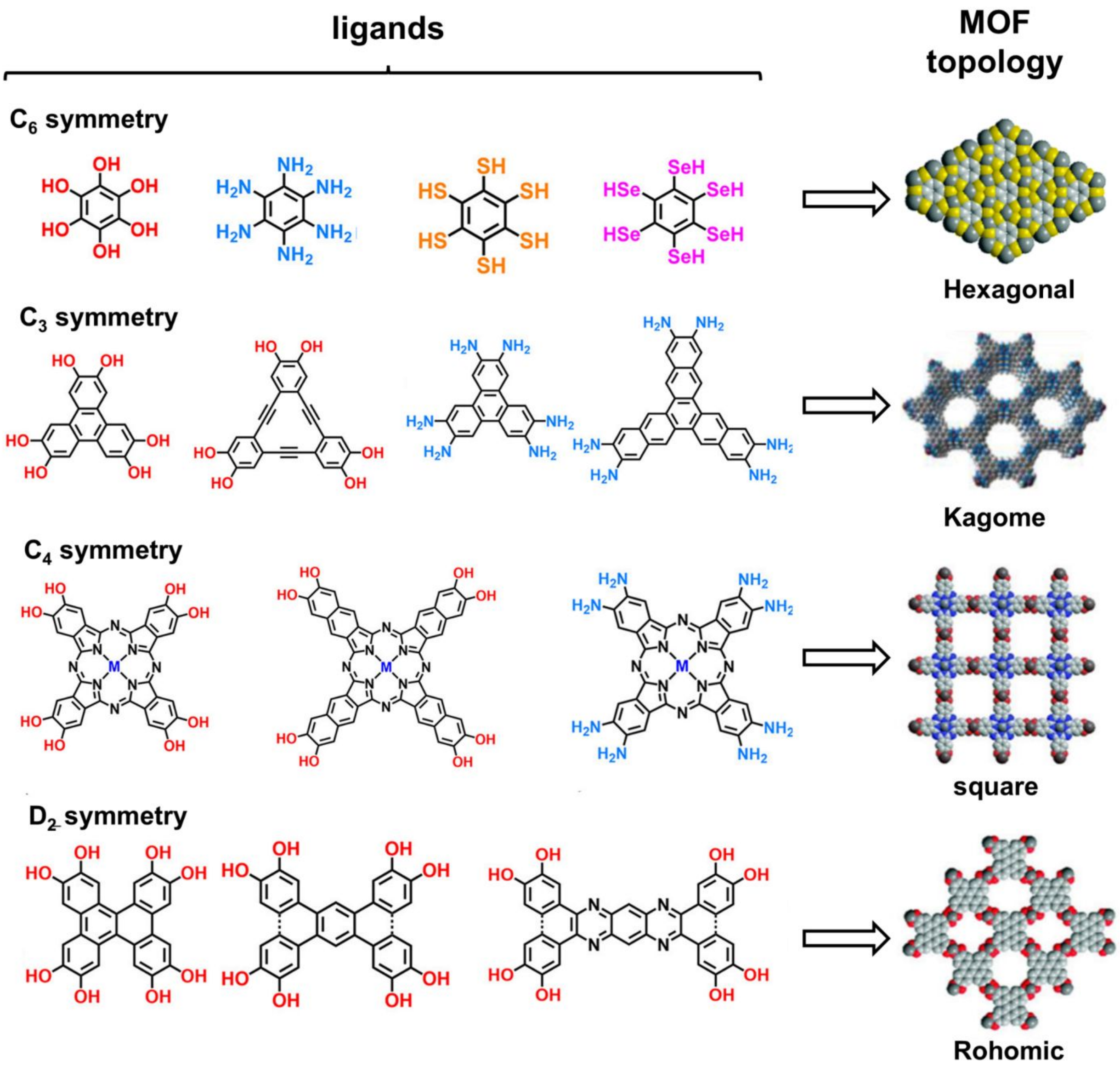

Figure S16. Typical topologies of layered conjugated MOFs with intrinsic electrical conductivity based on the symmetry of organic ligands.

2.4 SEM images of Mn-HHTP-kgm and Mn-HHTP-bex-d

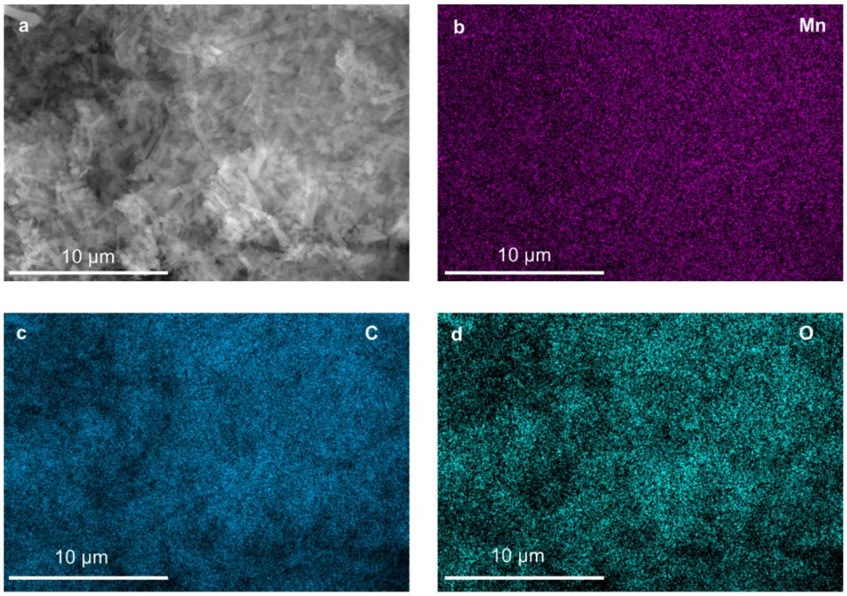

Figure S17. a) SEM image of $Mn_3(HHTP)_2$ MOF (Mn-HHTP-**kgm**) and corresponding energy dispersive X-ray spectroscopy (EDS) elemental mapping of b) Mn, c) C, and d) O.

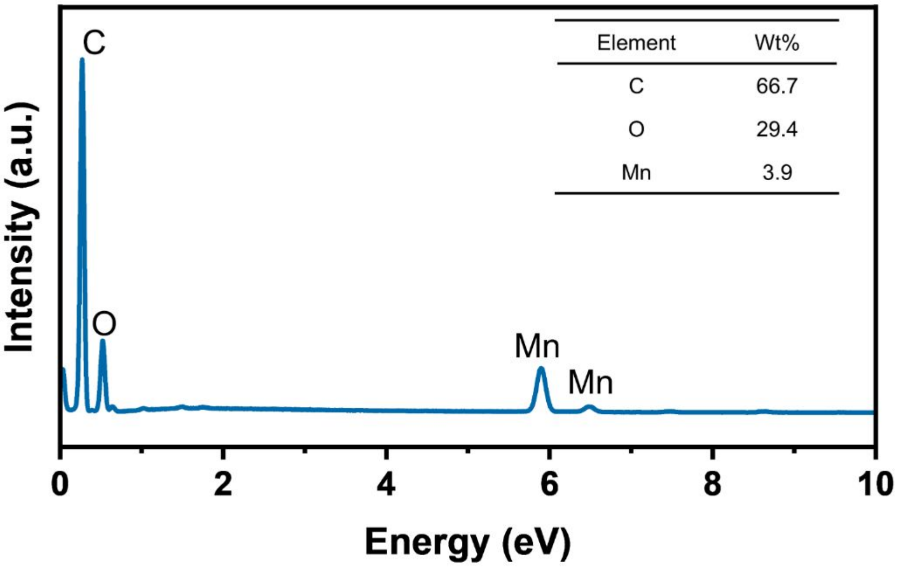

Figure S18. Point X-ray source elemental analysis of $Mn_3(HHTP)_2$ MOF (Mn-HHTP-**kgm**). MOF powder was coated with Au/Pd to reduce the charging.

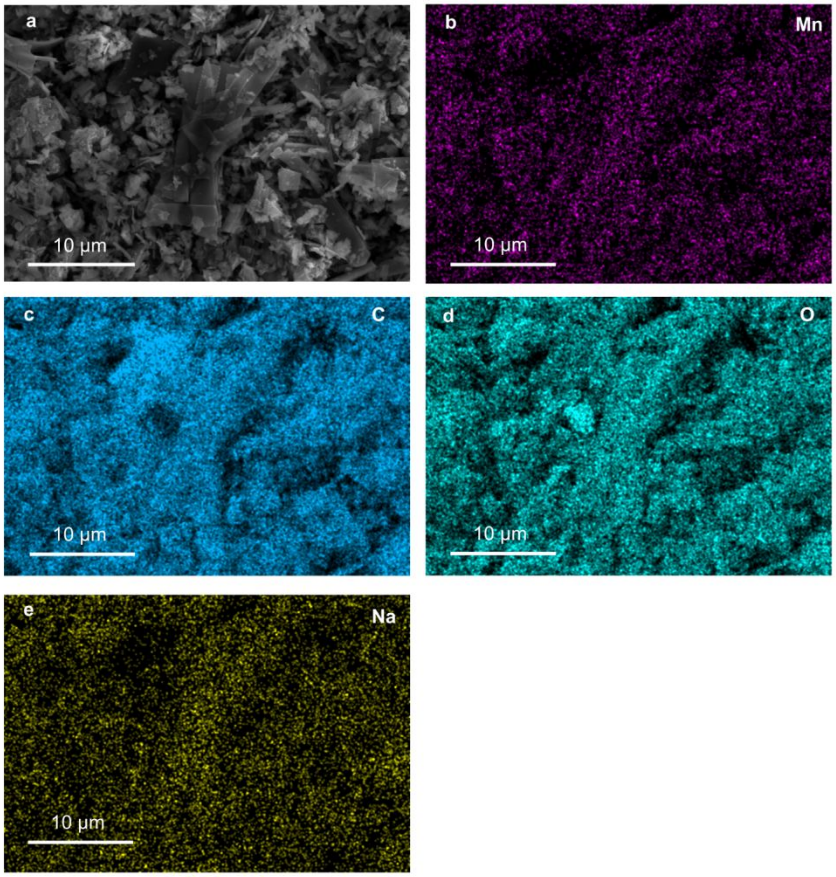

Figure S19. a) SEM image of Mn-HHTP-**bex-d** MOF and corresponding EDS elemental mapping of b) Mn, c) C, d) O, and e) Na.

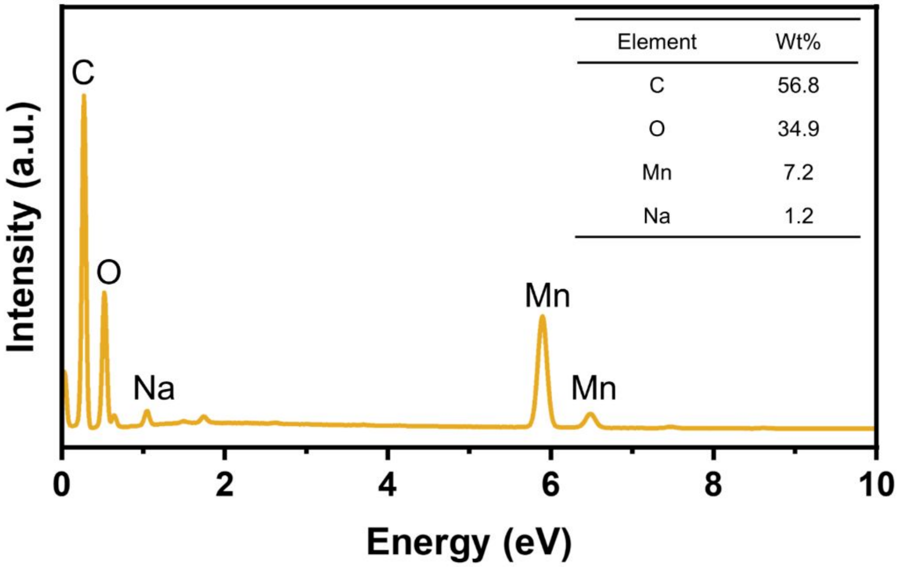

Figure S20. Point source elemental analysis of Mn-HHTP-**bex-d** MOF.

2.5 TEM image of Mn-HHTP-kgm

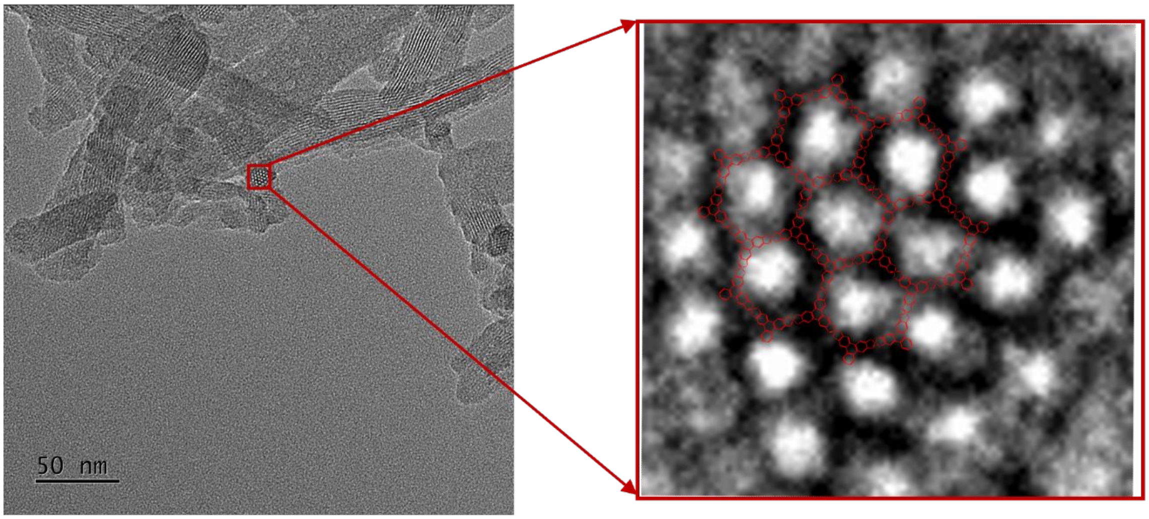

Figure S21. TEM images of $Mn_3(HHTP)_2$ MOF (Mn-HHTP-**kgm**).

2.6 Brunauer–Emmett–Teller (BET) Analysis

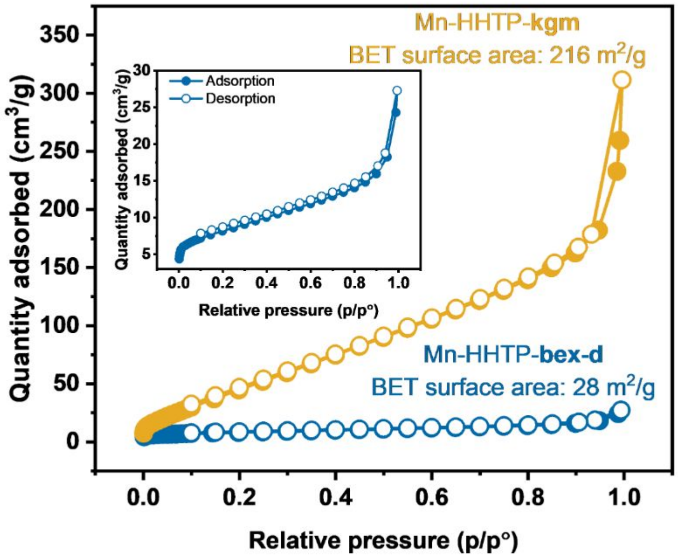

Figure S22. N_2 sorption isotherm at 77 K of Mn-HHTP-**kgm** and Mn-HHTP-**bex-d**.

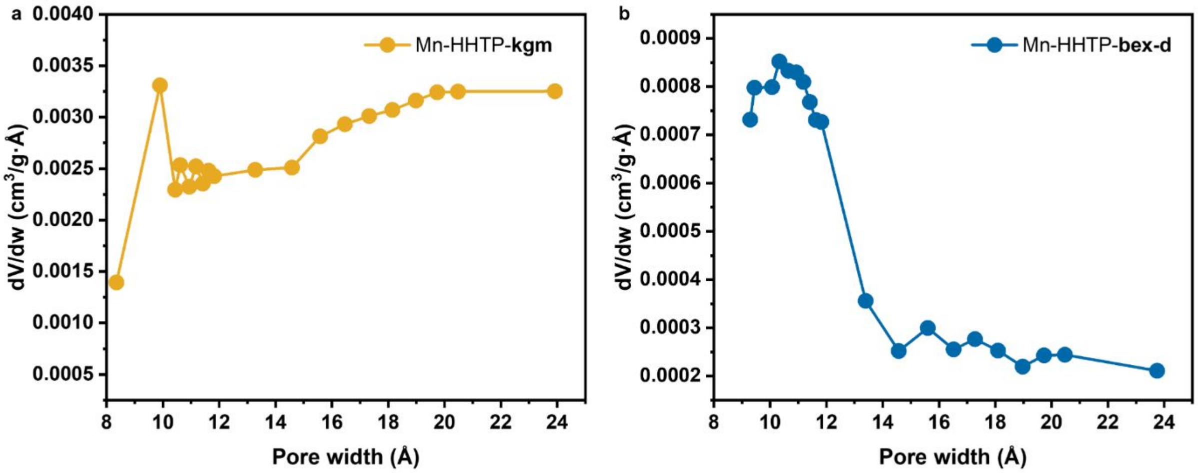

Figure S23. Pore size distribution based on Horvath-Kawazoe (H-K) method: a) Mn-HHTP-**kgm** and b) Mn-HHTP-**bex-d**.

2.7 EPR

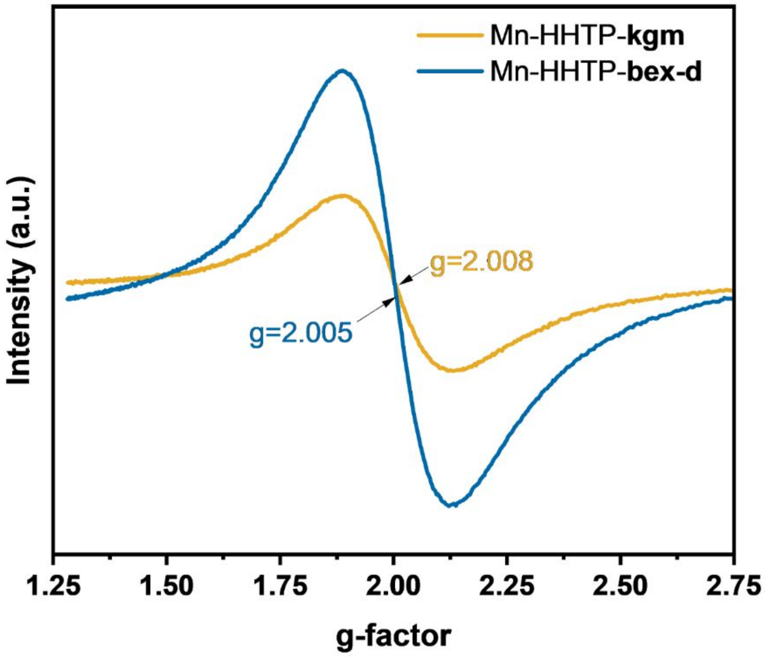

Figure S24. EPR spectra of Mn-HHTP-**kgm** and Mn-HHTP-**bex-d**.

2.8 XPS experiments

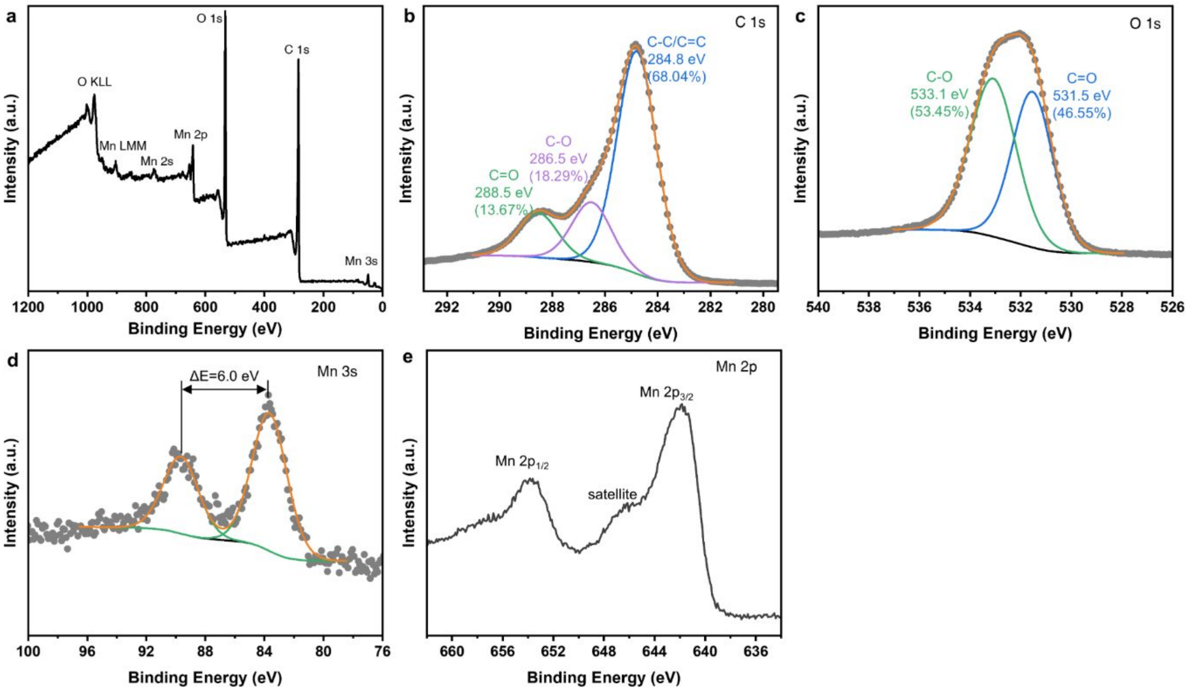

Figure S25. X-ray photoelectron spectra of Mn-HHTP-**kgm**: a) wide scan, b) C 1s, c) O 1s, d) Mn 3s, and e) Mn 2p.

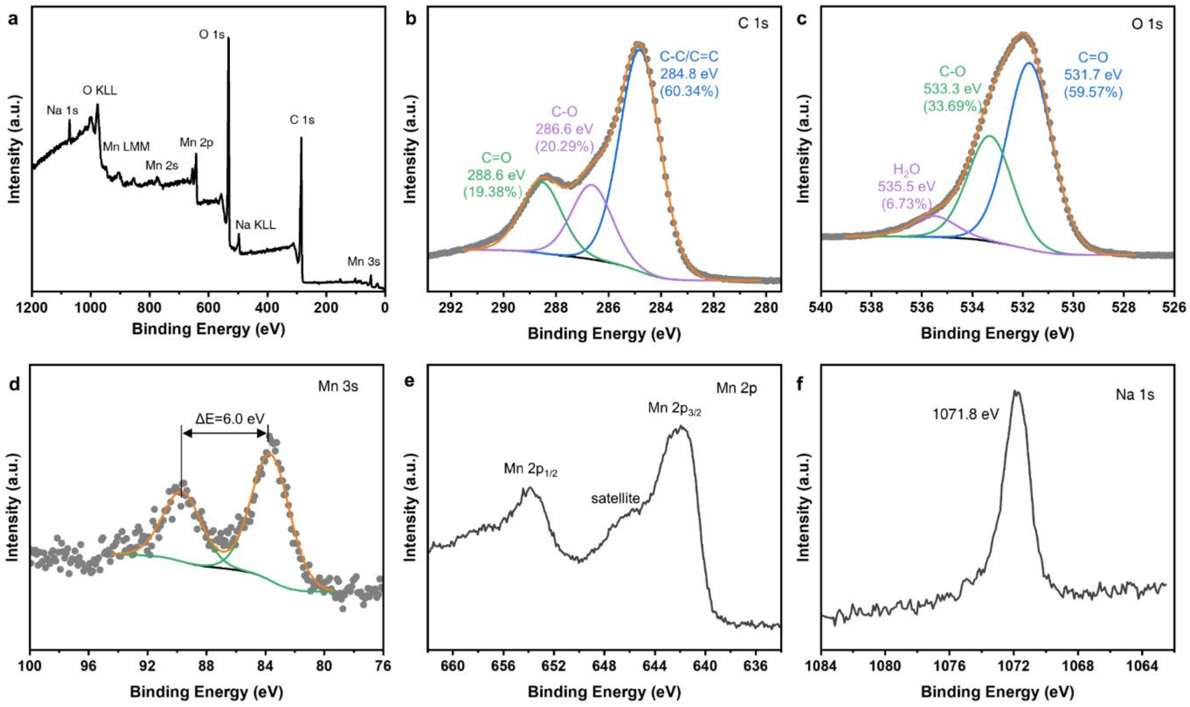

Figure S26. X-ray photoelectron spectra of Mn-HHTP-**bex-d** MOF: a) wide scan, b) C 1s, c) O 1s, d) Mn 3s, e) Mn 2p, and f) Na 1s.

2.9 FT-IR

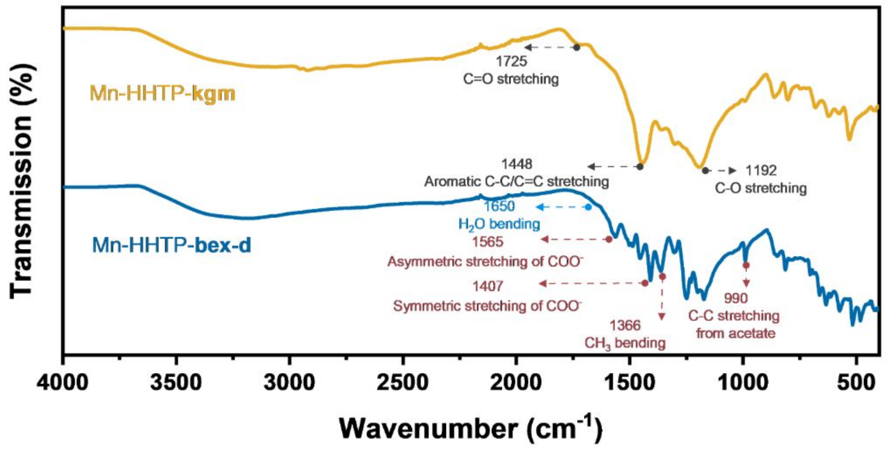

Figure S27. FT-IR curves of Mn(HHTP)-**kgm** and Mn-HHTP-**bex-d**.

2.10 Structural stability of Mn-HHTP-kgm and Mn-HHTP-bex-d

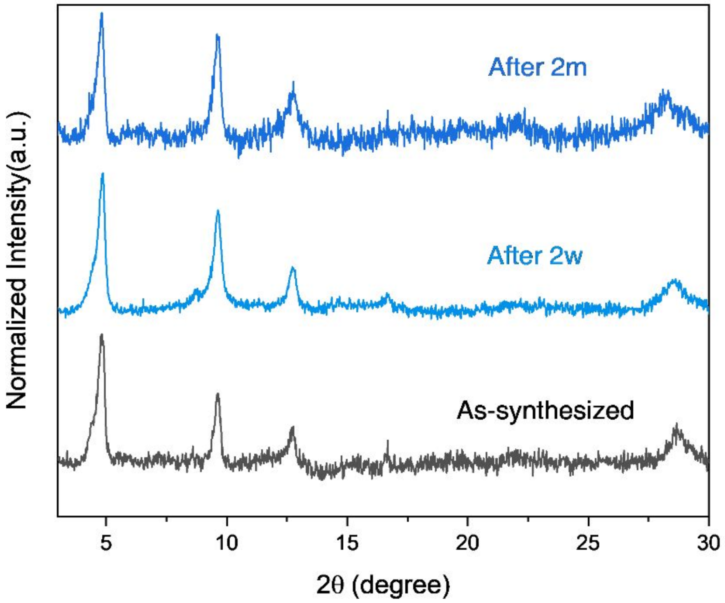

Figure S28. Structural stability of Mn-HHTP-**kgm** under room temperature and dry air. Crystallinity is maintained even after 2 months. It should be noted maintaining the structural stability of Mn-HHTP-**kgm** requires dry conditions. Mn-HHTP-**kgm** was found to be highly moisture-sensitive and tends to degrade quickly in a highly humid environment (**Figure S50**).

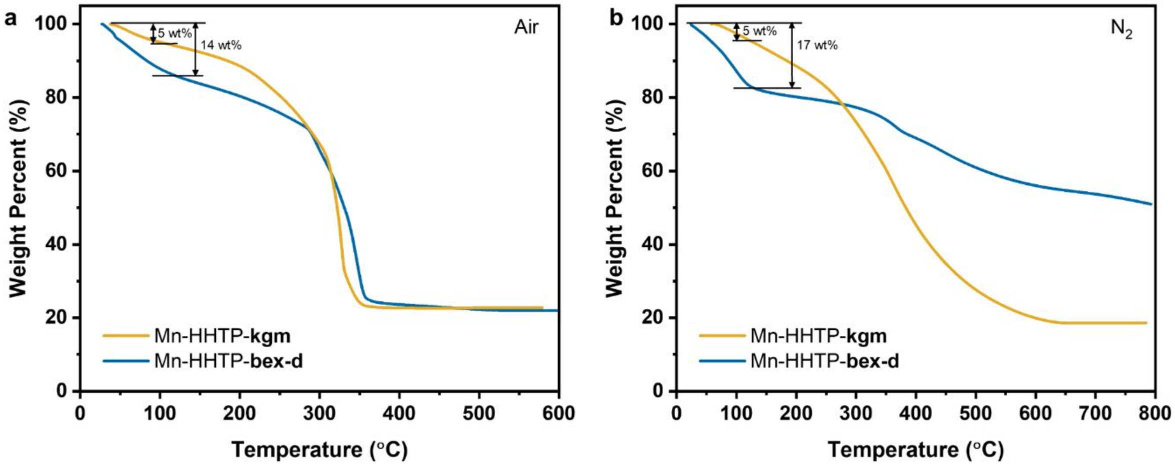

Figure S29. Thermogravimetric analysis (TGA) curve of Mn-HHTP-**kgm** and Mn-HHTP-**bex-d** in a) Air and b) N_2.

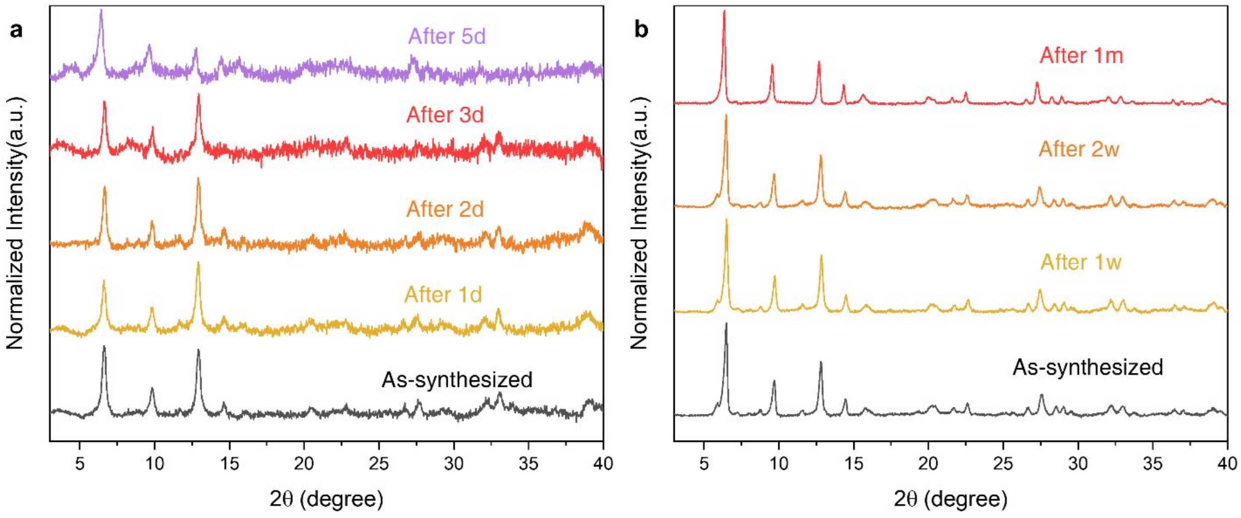

Figure S30. Structural stability of Mn-HHTP-**bex-d**: a) under ambient conditions (room temperature and air) and b) at low temperature of -22 °C. The crystallinity of Mn-HHTP-bex-d starts decreasing after 5 days under ambient conditions.

3. Computational Study for structure simulation and electronic band structure

Structure simulation and optimization were conducted using density functional theory (DFT) in the Cambridge Serial Total Energy Package (CASTEP)[1] module in Materials Studio 2025. Generalized gradient approximation (GGA) with Perdew-Burke-Emzerh (PBE) parametrization with Tkatchenko-Scheffler (TS)'s DFT correction was used with on-the-fly (OTFG) ultrasoft pseudopotentials. For Mn-HHTP-**kgm**, each modeled structure was energy minimized using 1×1×4 Monkhorst-Pack k-point mesh with a real-space mesh cut-off of 571.4 eV. For Mn-HHTP-**bex-d**, the SCXRD-resolved crystal structure was imported with the duplicated atoms with fractional occupancy (crystallographic disorders) removed, and then the built system including both unit cell and atom locations was further optimized using 4×1×2 k-point mesh with a real-space mesh cut-off of 598.7 eV. In all the DFT calculations for geometry optimization, the energy, force, stress and displacement convergence criteria were set as 1×10^{-5} eV, 0.03 eV/Å, 0.05 GPa and 0.001 Å, respectively.

The spin-polarized DFT calculations of the band structure, density of state, and electron density difference were carried out using the CASTEP[1] module in Materials Studio 2025. The exchange-correlation energy was described using the GGA-PBE functional with OTFG ultrasoft pseudopotentials. The Hubbard U correction was also adopted to the DFT energy calculations of the two MOF structures. The value of U parameter of Mn atom was set as 5.5 eV.[2] The kinetic energy cutoffs of electron wave functions were set as 571.4 and 598.7 eV for Mn-HHTP-**kgm** and Mn-HHTP-**bex-d**, respectively. The gamma-centered k-point grids 1×1×4 and 4×1×2 were used for Mn-HHTP-**kgm** and Mn-HHTP-**bex-d**, respectively. The convergence criterion for the self-consistent field calculation was set to 1×10^{-6} eV/atom.

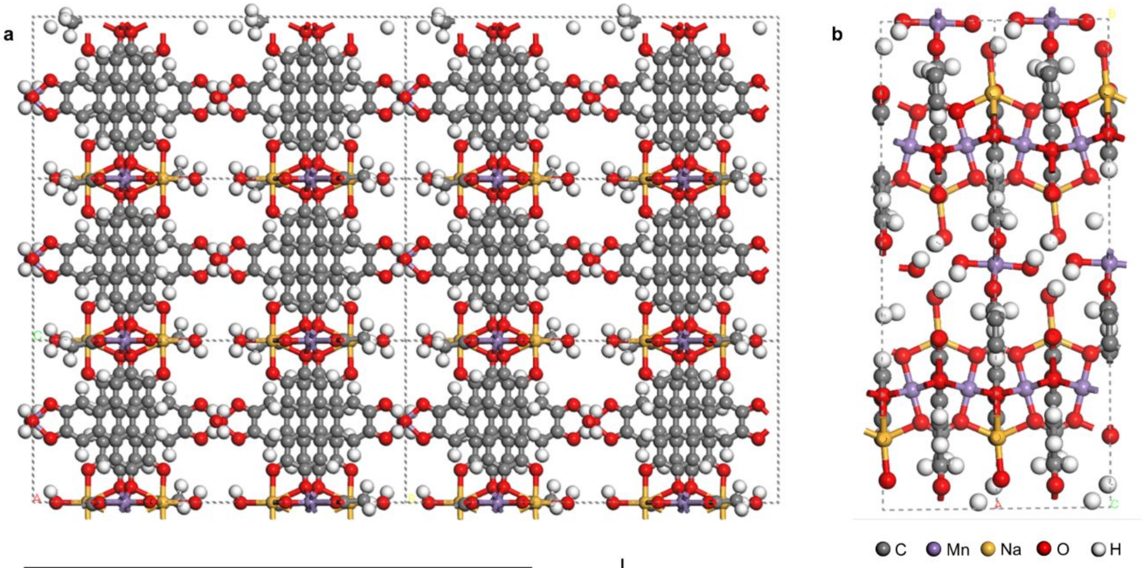

c

	SCXRD-resolved Structure	Optimized Structure
a	6.44 Å	6.49 Å
b	27.95 Å	27.84 Å
c	12.32 Å	12.09 Å
α	90°	90.04°
β	90°	90.02°
γ	90°	90.16°

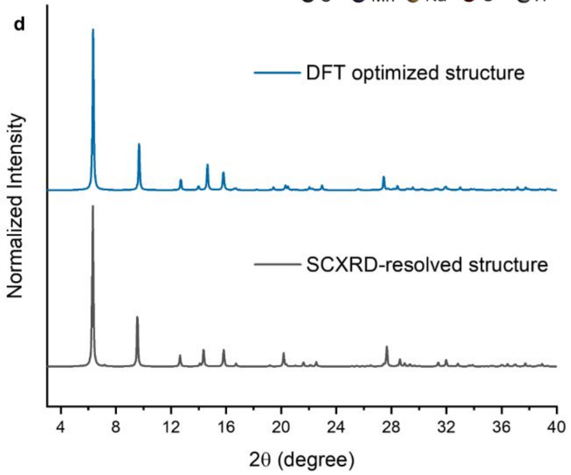

Figure S31. Structure of Mn-HHTP-**bex-d** after DFT optimization for spin-polarized energy calculations a) along a-axis and b) along c-axis. c) Lattice parameter comparison between SCXRD-resolved structure and DFT optimized structure. d) Simulated PXRD pattern comparison between SCXRD-resolved structure and DFT optimized structure.

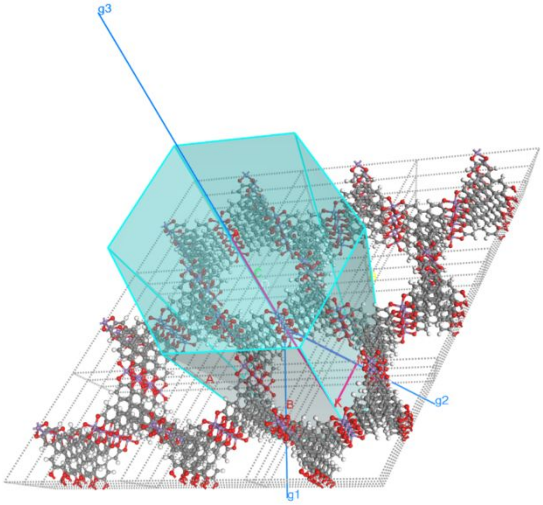

Figure S32. The first Brillouin zone (box with blue lines) and high-symmetry K-points (Γ, M, K, and A) for Mn-HHTP-**kgm**.

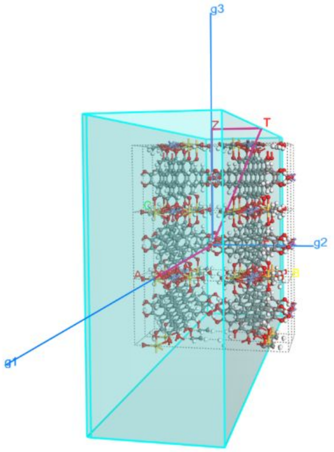

Figure S33. The first Brillouin zone (box with blue lines) and high-symmetry K-points (Γ, X, T, and Z) for Mn-HHTP-**bex-d**.

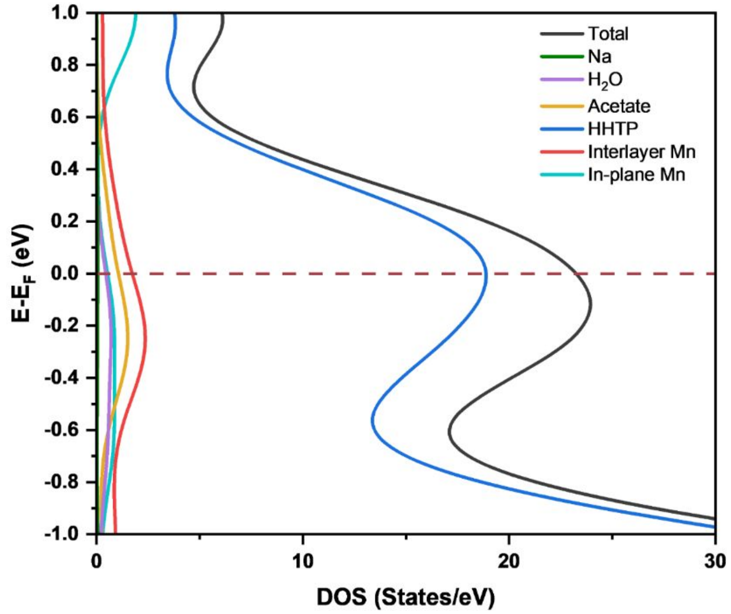

Figure S34. Project density of states (PDOS) analysis based on groups for Mn-HHTP-**bex-d**.

4. Electronic conductivity and electronic structure

4.1 Room-temperature electronic conductivity measurements

A non-destructive four-point probe method with a commercial collinear pin system was used for conductivity measurement of materials. First, the as-prepared powder materials were compressed into a cylinder pellet in 6 mm diameter under 1000 psi for 8 minutes by using a pellet press (Desktop Pellet Press, Across International). Then, the bulk electrical conductivity of the pellet was measured using a four-point probe (Pro4 Resistivity Test System equipped with a high-resistivity probe head, Signatone) with a uniform 1.5875 mm space between the probes. According to the *V/I* value directly obtained from 4-point probe measurement, we calculated the bulk conductivity in Siemens per centimeter using the following equation:

$$\sigma = \frac{I}{V}\frac{1}{2\pi s F_i}$$

where *I* (A) is current, *V* (V) is the voltage, *s* (cm) is distance of between the probes (1.5875 mm in this case), F_i is the correction factor accounting for the diameter and thickness of the pellet. The electronic conductivity measurements were conducted under ambient conditions (~298 K, ~35% RH).

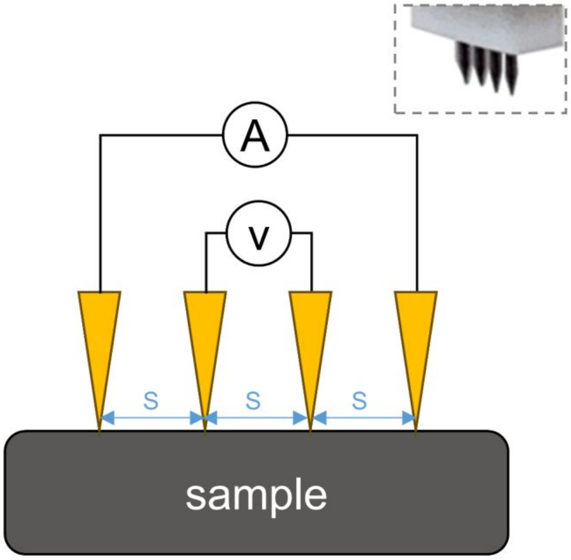

Figure S35. The non-destructive four-point probe method with a commercial collinear pin system (inset: an image of an actual commercial four-probe pin system) electronic conductivity under ambient conditions.

4.2 Variable temperature electronic conductivity data

Variable-temperature electrical conductivity measurements were conducted using Physical Property Measurement System (PPMS) from Quantum Design. Due to the requirement of extremely low temperatures, the temperature-dependent conductivity data were collected under vacuum. The as-prepared powder materials were compressed into a square pellet in 6 mm side length under 1000 psi for 8 minutes by using a pellet press (Desktop Pellet Press, Across International). Au wire with a diameter of 0.001 in and carbon paste were used for four-probe electrode configuration, as shown in **Figure S36**. The four-probe (Au wires) were connected to the resistivity puck for Physical Property Measurement System (PPMS) from Quantum Design using conductive carbon paste. The DC resistance mode of PPMS was used to collect resistivity data of the pelletized sample. The bulk conductivity in Siemens per centimeter using the following equation:

$$\sigma = \frac{1}{R}\frac{1}{2\pi s F_i}$$

where R (Ω) is the measured resistivity, s (cm) is spacing between the probes, F_i is the correction factor accounting for the diameter and thickness of the pellet. For approximation, an average value of spacings between different probes was used for calculation.

Compared to the commercial pin system, the parallel wire configuration introduces contact resistance at the paste-sample interface and unavoidable geometric spacing errors. These experimental constraints systematically induce the measured electronic conductivity to be slightly lower than that measured using the non-destructive pin system (**Figure 4a**).

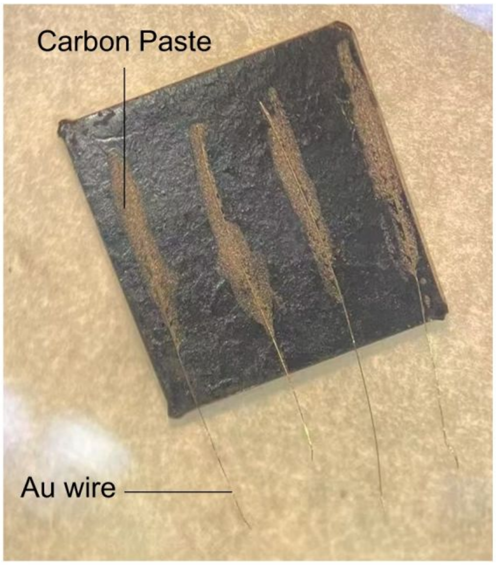

Figure S36. A custom-built parallel four-point probe configuration using gold wire and conductive paste on a square pellet with a 6 × 6 mm for variable-temperature electronic conductivity measurements.

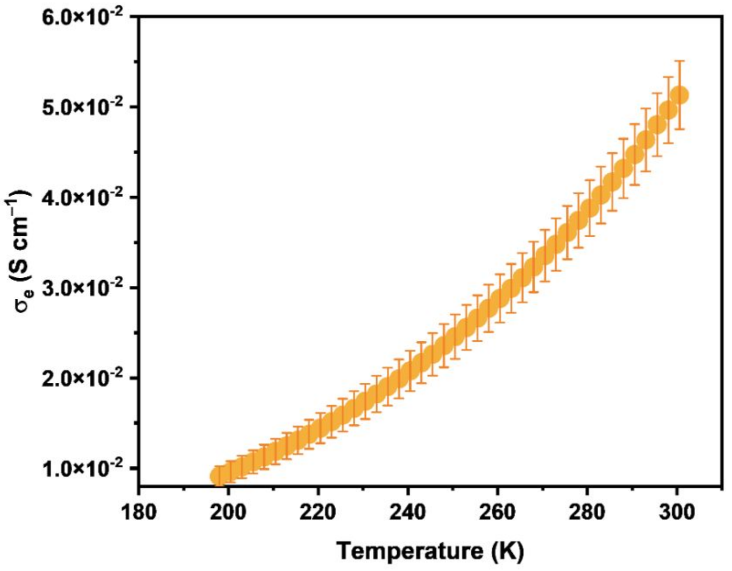

Figure S37. The variable-temperature electrical conductivity of Mn-HHTP-**kgm**.

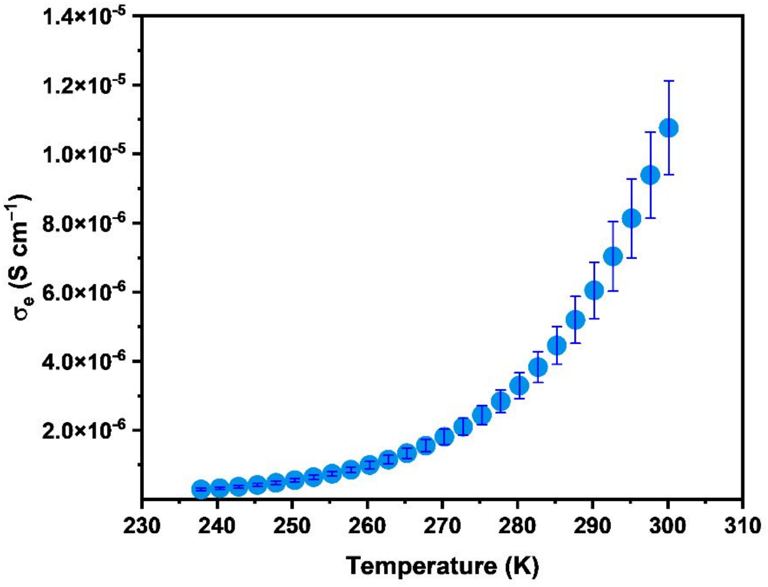

Figure S38. The variable-temperature electrical conductivity of Mn-HHTP-**bex-d**.

4.3 Room-temperature electrical conductivity comparison

Table S4. Room-temperature electrical conductivity values of some representative reported electrically conductive MOFs.

Materials	Topology	Room-temperature electrical conductivity ($S\ cm^{-1}$)	Method	Ref.
**Mn-HHTP-kgm**	**kagome**	**0.84**	**4-probe Pellet**	**This work**
$Ni_3(HITT)_2$	kagome	4.5	4-probe Pellet	3
$Cu_3(HITT)_2$	kagome	0.05	4-probe Pellet	3
$Cu_3(HITP)_2$	kagome	0.75	4-probe Pellet	4
$Cu_3(HHTP)_2$	kagome	0.007	2-probe Pellet	5
		0.70	Single Crystal	6
$Ni_3(HHTP)_2$	kagome	0.1	4-probe Pellet	7
Cu-HHTC	kagome	1.97×10^{-3}	4-probe Pellet	8
$Ni_3(HITP)_2$	kagome	2	2-probe Pellet	9
$Cu_3(HTB)$	hexagonal	1580	4-probe Film	10
$Fe_3(PTC)$	hexagonal	10	4-probe van der Pauw	11
$Cu_2(OHPTP)$	rhombic	0.1	4-probe van der Pauw	12
Ni-BHT	kagome	0.15	2-probe Pellet/Film	13
		1.6×10^{-2}	4-probe van der Pauw (Nanosheet)	14
$Ni_3(HITAT)_2$	kagome	0.044	2-probe Pellet	15
$Ni_3(HITBim)_2$	kagome	5×10^{-4}	2-probe Pellet	15
$Ni_3(HATI\_C1)_2$	kagome	0.011	4-probe van der Pauw	16
$Ni_3(HATI\_C3)_2$	kagome	4.5×10^{-3}	4-probe van der Pauw	16
$Ni_3(HATI\_C4)_2$	kagome	9×10^{-4}	4-probe van der Pauw	16
CuPc-O-Ni	square	0.046	4-probe Pellet	17
CuPc-O-Cu	square	0.093	4-probe Pellet	17
CuPc-O-Zn	square	0.029	4-probe Pellet	17
$NiPc-NiO_4$	square	4.8×10^{-7}	2-probe Pellet	18

NiPc-Ni	square	7.22×10^{-5}	4-probe Pellet	19
NiPc-Cu	square	0.0143	4-probe Pellet	19
NiNPc-Ni	square	0.0178	4-probe Pellet	19
NiNPc-Cu	square	0.0313	4-probe Pellet	19
PcNi-Co-O	square	1.1×10^{-8}	4-probe Pellet	20
$Cu_3(HHB)_2$	kagome	7.3×10^{-8}	4-probe van der Pauw	21
$Cu_3(HIB)_2$	kagome	3.23×10^{-2}	4-probe Pellet	22
HATN-O-Zn	kagome	1.23×10^{-7}	4-probe Pellet	23
Cu-DHHBTN	dual pore-honeycomb	0.21	4-probe Pellet	24
Sm-HHTP	pseudo kagome	4.3×10^{-5}	4-probe Pellet	25
Eu-HHTP		3.0×10^{-5}	4-probe Pellet	25
Gd-HHTP		7.6×10^{-6}	4-probe Pellet	25
Tb-HHTP		2.7×10^{-6}	4-probe Pellet	25

4.4 Electronic Structure

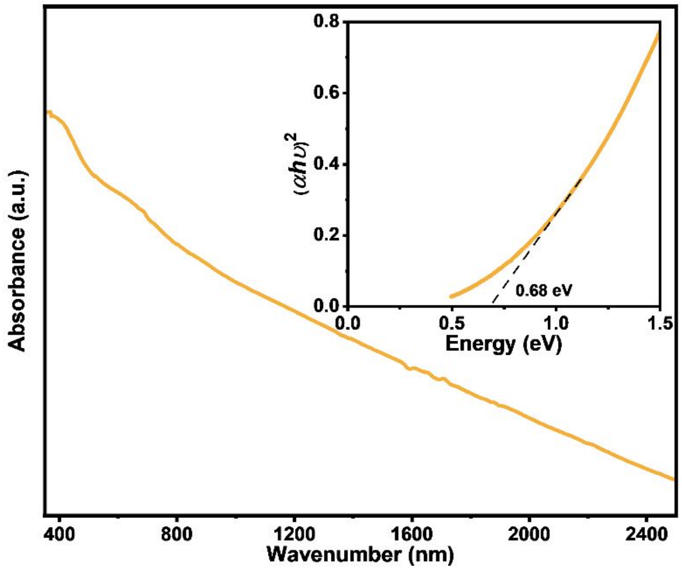

Figure S39. UV-vis -NIR spectra of Mn-HHTP-**kgm** (inset: corresponding Tauc plot).

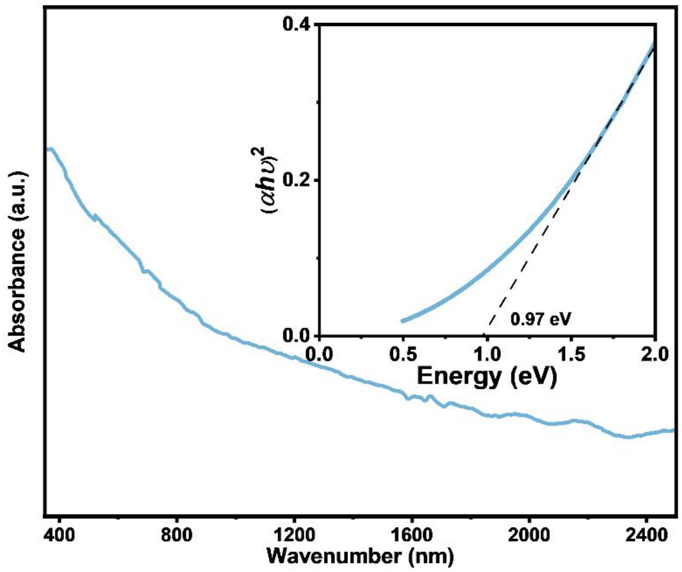

Figure S40. UV-vis -NIR spectra of Mn-HHTP-**bex-d** (inset: corresponding Tauc plot).

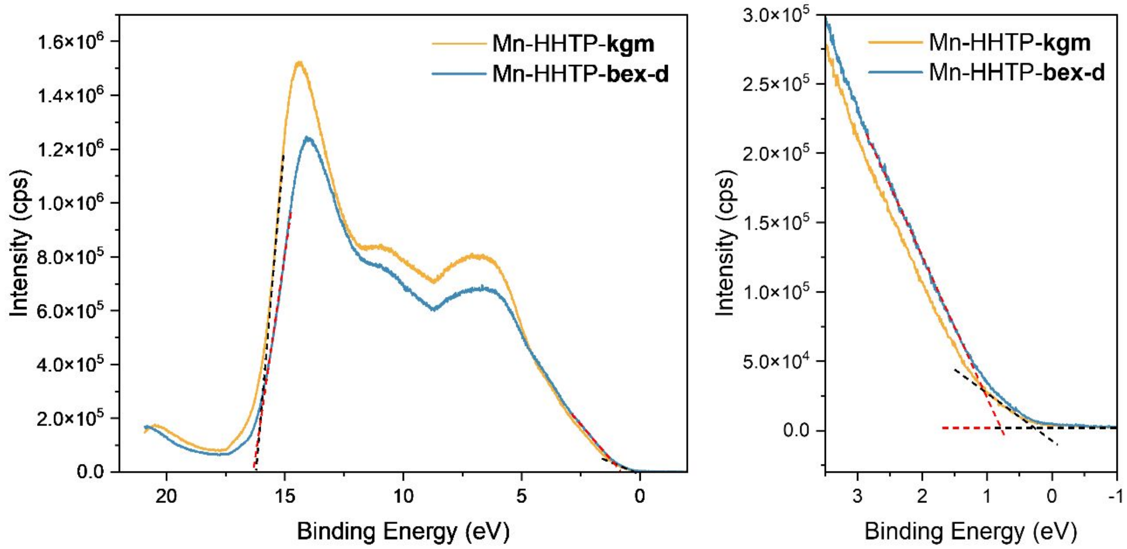

Figure S41. UPS spectra of Mn-HHTP-**kgm** and Mn-HHTP-**bex-d**.

4.5 Hall measurements

The MOF powder sample was first pressed into a square pellet with a side length of 6 mm and a thickness of around 0.5~0.6 mm under 1000 psi for 8 minutes by using a pellet press (Desktop Pellet Press, Across International). The van der Pauw configuration method was employed, and all direct current (DC) Hall measurements were conducted using the MeasureLink M91 FastHall system connected with the Quantum Design PPMS system. Conductive silver paste and Au wire were used to connect the MOF pellets and the sample puck. In the Hall effect measurements, MOF samples were tested with a magnetic field vertical to the flat surfaces with a magnetic field sweeping from 4 to -4 T.

The Hall mobility (μ) of the MOF was calculated according to the following equation:

$$\mu = \rho \cdot \frac{V_{Hall} t}{I \cdot B}$$

Where V_{Hall} is the Hall voltage, B is the applied magnetic field, t is the sample thickness, I is the current (0.4 mA in this case), and ρ is the directly measured electrical conductivity.

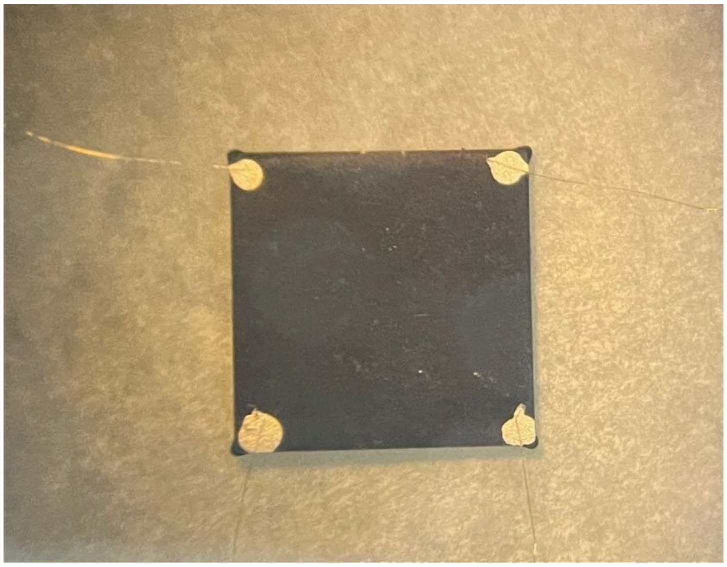

Figure S42. An example of the Van der Pauw configuration of a square pellet with a size of 6 × 6 mm for Hall measurement.

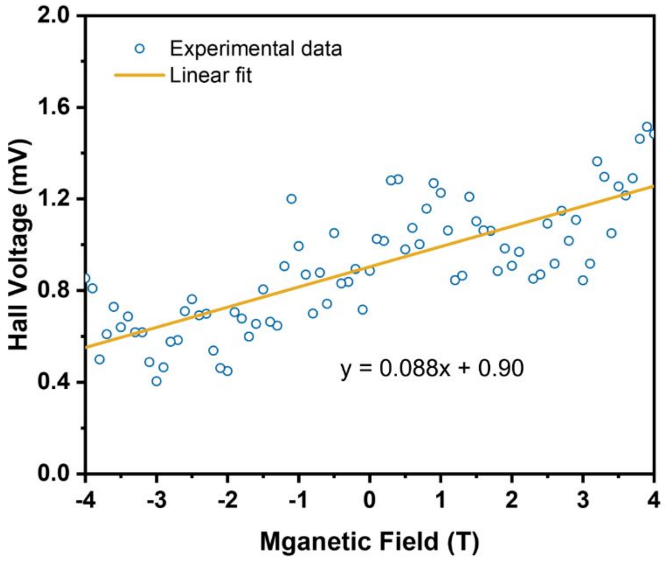

Figure S43. Hall effect for Mn-HHTP-**kgm**: magnetic field-dependent Hall voltage.

The nonzero intercept at $B = 0$ in **Figure S43** most likely arises from a systematic voltage offset due to factors such as contact asymmetry, longitudinal resistance pickup, and thermoelectric effects. In our measurements and manufacturing process, the electrodes were manually prepared with silver paste rather than wire bonding, which likely introduced unavoidable asymmetry for the van der Pauw method. In addition, Hall measurements on pelletized polycrystalline samples with relatively low conductivity (10^{-2}–10^{-1} S cm^{-1}) are inherently challenging because the carrier mobility is close to the detection limit of our Hall measurement setup, yielding noisy data.

5. Proton conductivity measurements

Proton conductivities of the Mn-HHTP materials were measured through blocking-electrode method by using a potentiostat (VMP3 potentiostat, BioLogic or Interface 1010, Gamry Instruments). Similar to sample preparation for 4-point probe measurement, sample were pressed into a die under 1000 psi for 8 minutes to obtain uniform pellets (1−2 mm in thickness, 6.0 mm in diameter). To enhance contact, the top and bottom sides of the sample pellet were coated with gold through physical deposition. The sample pellet was loaded into a custom two-electrode cell between two stainless steel sheets and was then inserted in a humidity chamber maintained at 98% relative humidity (RH) for 24 hrs before temperature-variable proton conductivity measurements. The Nafion (Nafion-117, Dupont, Fuel Cell Store) served as blocking electrodes for proton-conductivity measurements. Electrochemical Impedance Spectroscopy (EIS) was performed at AC mode with no bias at frequencies from 1 MHz to 1 Hz. Proton conductivity (σ_H) can be calculated by the following equation:

$$\sigma_H = \frac{L}{R_p A}$$

Where σ is proton conductivity (S cm^{-1}), L (cm) is the thickness of pellet, R_p (Ω) is the measured resistance for proton transport, and A is the geometric area of pellet (cm^2). Due to the superprotonic conductivity of Nafion membrane, its contribution to the resistance (R_p) can be neglected.

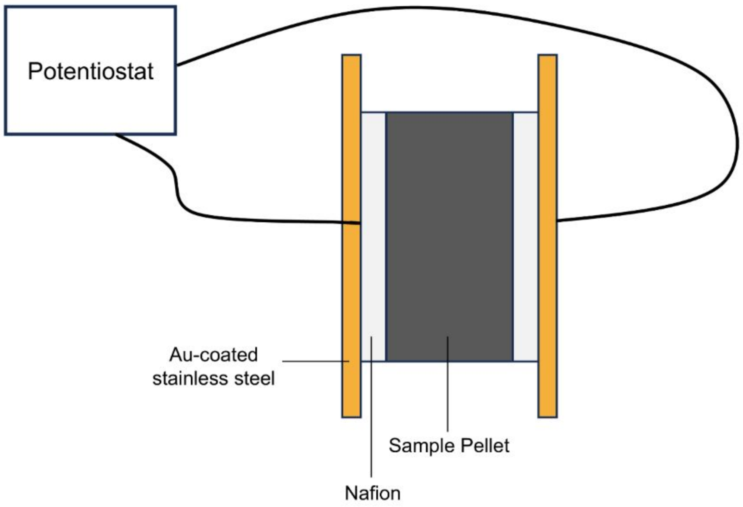

Figure S44. Schematic illustration of the two-blocking electrode method for proton conductivity measurement.

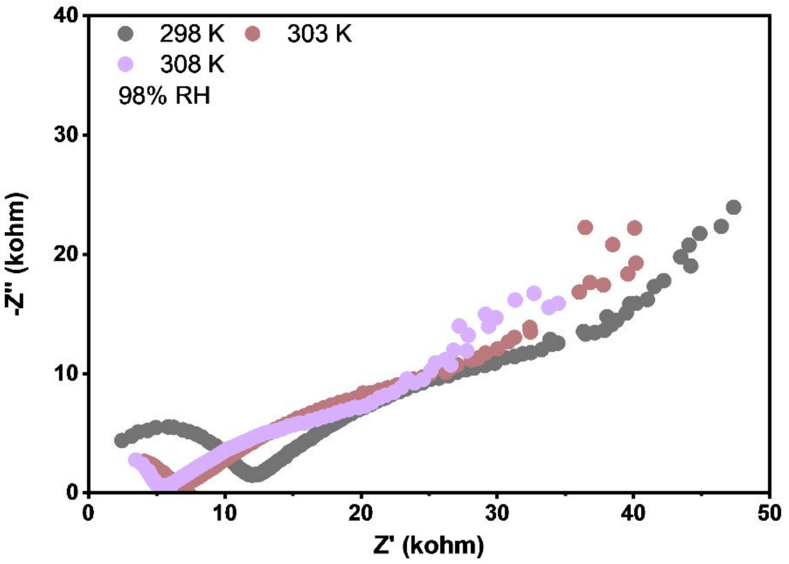

Figure S45. Representative Nyquist plots measured with 98% RH at relatively low temperatures (298, 303, and 308 K) for pelletized Mn-HHTP-**bex-d**. These selected Nyquist plots exhibit an additional semicircle in the intermediate frequency range, which may arise from grain-boundary contributions. As the temperature increases, the second semicircle feature becomes less noticeable.

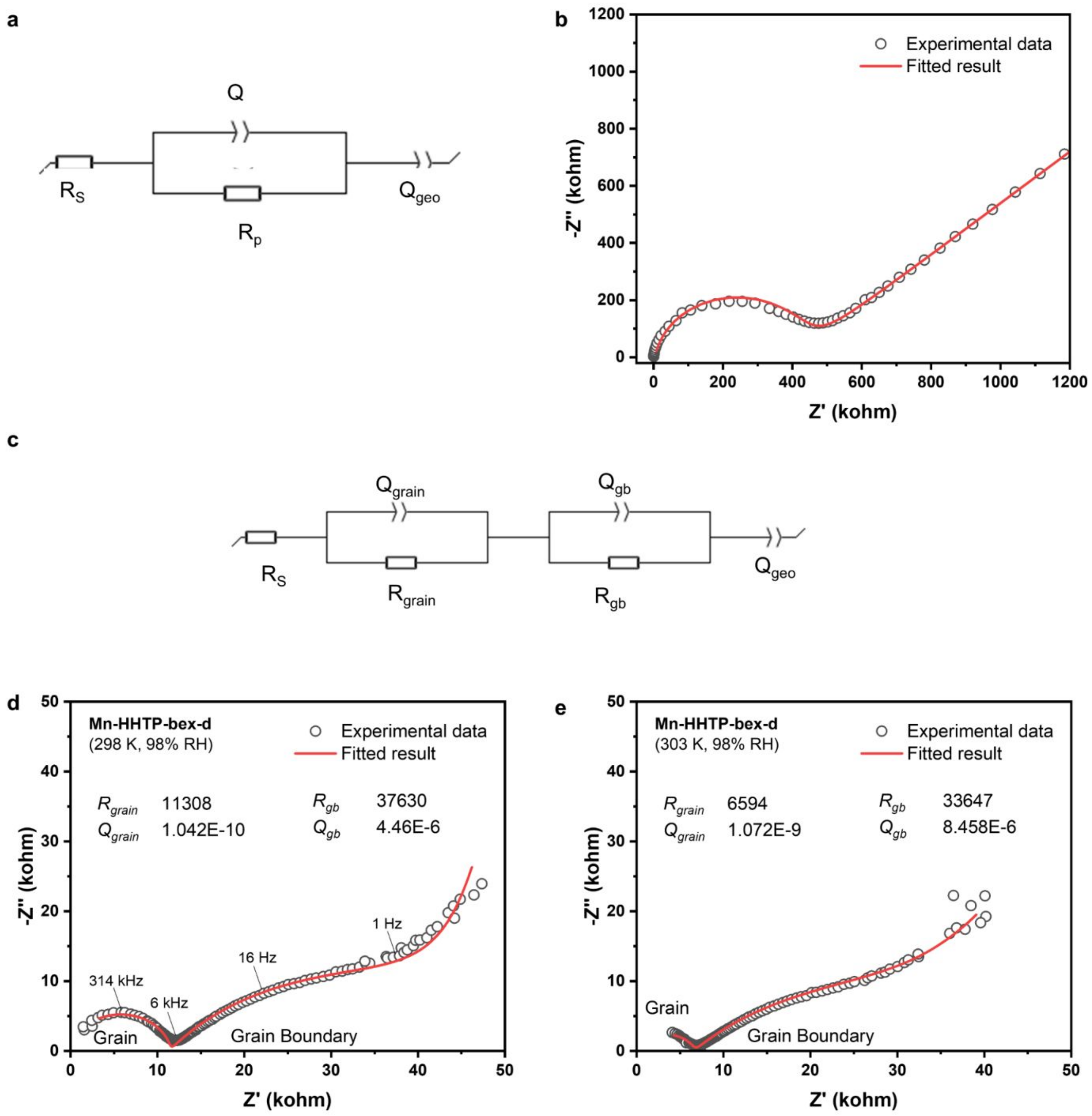

Figure S46. a) Equivalent circuit model for proton conductivity measurements. R_s, Q, R_p, and Q_{geo} represents a series resistance, a constant phase element for non-ideal and non-homogeneous surface behavior, proton transport resistance, and a constant phase element for the geometric proton diffusion behavior, respectively. b) An example of Nyquist plot and the fitted result using the established model in (a). c) Equivalent circuit model for proton conductivity measurements considering the grain boundary effects. Q_{grain} and R_{grain} represent the grain contributions to the proton conduction. Q_{gb} and R_{gb} represent the grain boundary effect. d) and e) examples of Nyquist plot and the fitted results using the established model in (c). Frequencies of a few relevant data

points are indicated in (d). By comparing the best-fit parameters of R_{grain} and R_{gb}, the values of R_{grain} are lower than the corresponding R_{gb} values in (d) and (e).

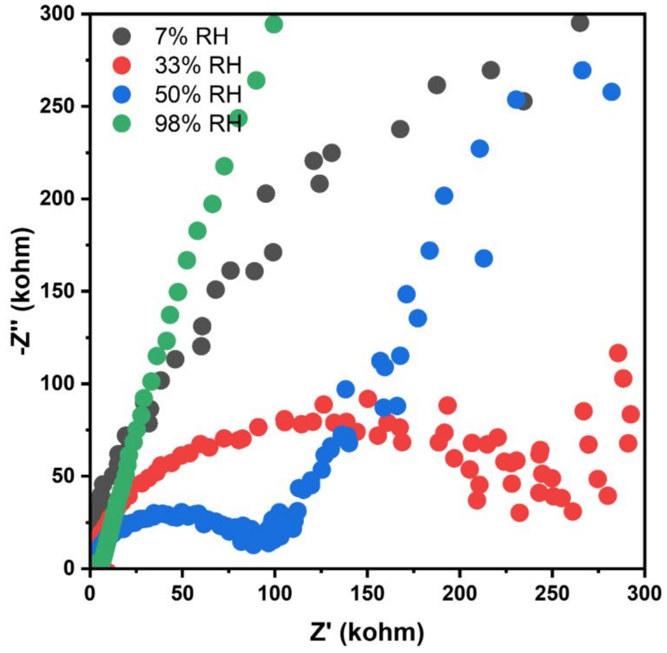

Figure S47. Nyquist plots of Mn-HHTP-**bex-d** at various relative humidities at room temperature.

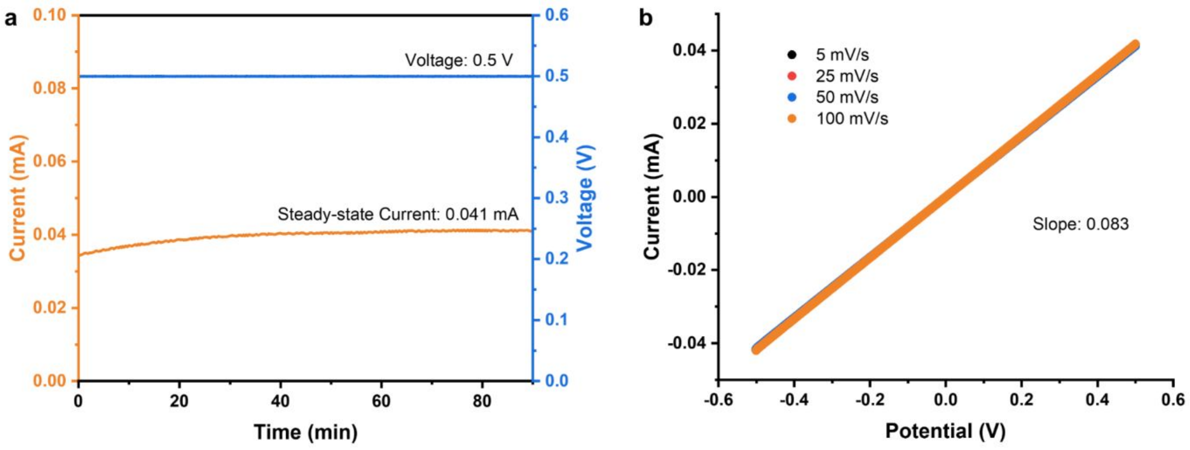

Figure S48. Electrical conductivity measurements of a pressed pellet of Mn-HHTP-bex-d material: (a) Chronoamperometry test at 0.5 V and (b) I-V determinations in the range of -0.5 to 0.5 V at different scan rates (5, 25, 50 and 100 mV/s). The I/V values calculated from the steady-state chronoamperometry current and I-V measurements are highly consistent, suggesting that proton conduction contributes minimally to the measured electronic conductivity of Mn-HHTP-**bex-d** under ambient conditions (~298 K, ~35% RH).

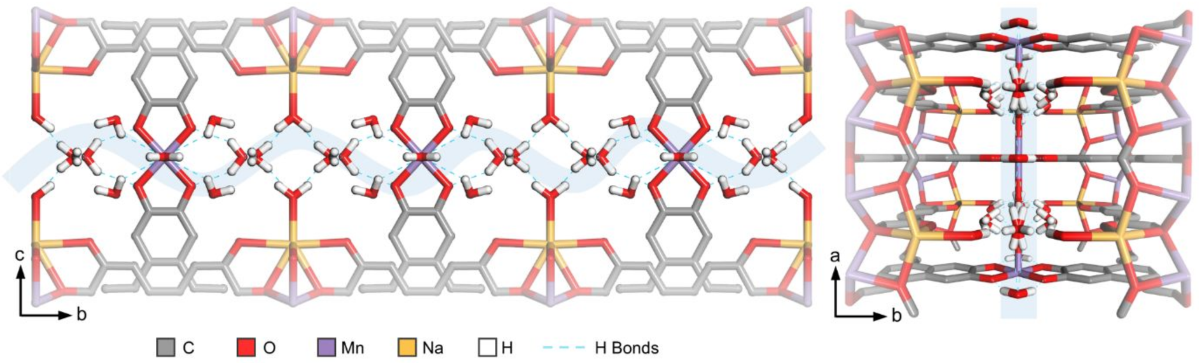

Figure S49. All SCXRD-resolved arrangements of water molecules and hydrogen-bonding networks within the channels of Mn-HHTP-**bex-d**, viewed along the *a* and *c* axes. All water molecules with partial occupancies are presented.

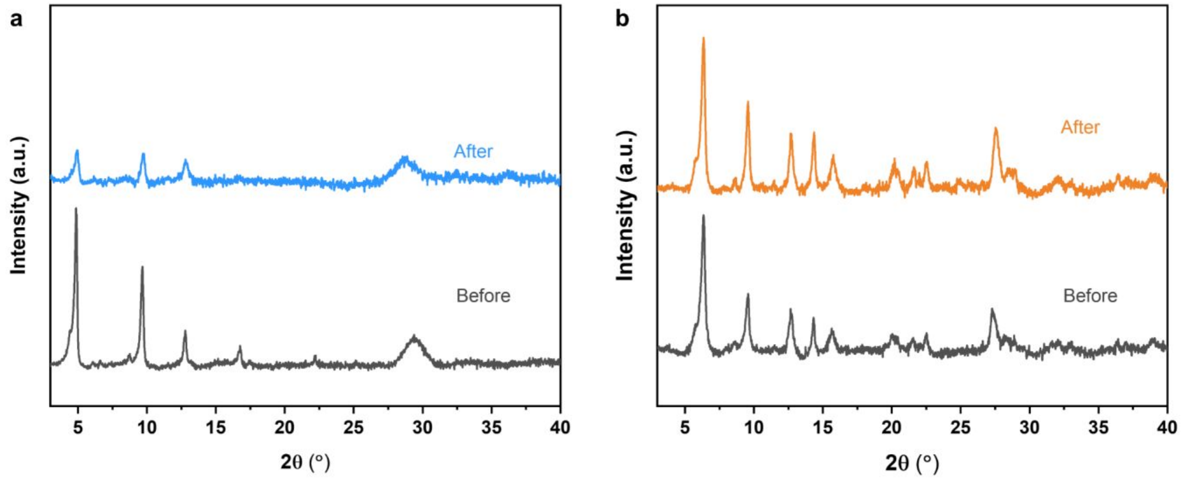

Figure S50. Structural stability of (a) Mn-HHTP-**kgm** and (b) Mn-HHTP-**bex-d** after 5-hour exposure to hot and humid air (60 °C, 98% RH). Mn-HHTP-**kgm** is very moisture-sensitive, whereas Mn-HHTP-**bex-d** exhibits noticeably improved tolerance toward moisture and humidity. Even though Mn-HHTP-**kgm** showed prolonged structural stability at dry conditions, it could lose crystallinity even faster than Mn-HHTP-**bex-d** when exposed to humid air.

Table S5. Dual Conductivity Performance Comparison

Material	Electronic Conductivity (unit: S/cm)*	Proton Conductivity (RH, T) (unit: S/cm)	Ref.
**Mn-HHTP-kgm**	**8.4×10^{-1}**	**3.6×10^{-7} (98% RH, 298 K)**	**This work**
**Mn-HHTP-bex-d**	**2.4×10^{-5}**	**4.5×10^{-5} (98% RH, 298 K)**	**This work**
Cu_3TABC_2	6×10^{-2}	1.6×10^{-5} S/cm (in water, 298 K)	26
Zn_3TABC_2	2.2×10^{-4}	1.9×10^{-5} (in water, 298 K)	26
$[(CH_3)_2NH_2][In(H_4TTFOC)]$	1.69×10^{-8} (303 K)	1.30×10^{-2} (98% RH, 303 K)	27
H_{12}-Fe_2-$DOBDP_3$	8.3×10^{-7} (293 K)	1.6×10^{-4} (hydrated, 293 K)	28
TTFTP-La	7.2×10^{-6}	4.9×10^{-5} (95% RH, 298 K)	29
Zn-HHTP-H_2O	4.6×10^{-2} (293 K)	1.1×10^{-6} (95% RH, 293 K)	30
Ni-BAND	2.4×10^{-1}	9×10^{-2} (> 90% RH, 298 K)	31
2D-Co-NS-PBS ($[Co_7(OH)_6(H_2O)_3(C_4H_4O_4)_4$ intercalated with PBS)	3.84×10^{-4} (398 K)	2.02×10^{-5} (99% RH, 398 K)	32
$Cu(INA)_2(H_2O)_4$	5×10^{-8} (400 K)	2.26×10^{-5} (98 % RH, 298 K)	33
Cu_3HHTT_2	10^{-7}	2.54×10^{-8} (70% RH, 298 K)	34
$[Cu_3(\mu_3\text{-}OH)(C_4H_2N_2O_2)_3(H_2O)] \cdot 2C_2H_5OH \cdot 4H_2O$	9.39×10^{-9} (353 K)	6.39×10^{-7} (98% RH, 353 K)	35

* Unless specified otherwise, the reported electrical conductivities were measured at room temperature (~298 K).

6. References

1. Clark, S. J.; Segall, Mathew D.; Pickard, Chris J.; Hasnip, Phil J.; Probert, Matt I. J.; Refson, Keith; Payne, Mike C. First Principles Methods Using CASTEP. *Zeitschrift für kristallographie-crystalline materials* **2005***, 220* (5-6), 567−570.

2. Zhang, Z.; Valente, D. S.; Shi, Y.; Limbu, D. K.; Momeni, M. R.; Shakib, F. A. In Silico High-Throughput Design and Prediction of Structural and Electronic Properties of Low Dimensional Metal−Organic Frameworks. *ACS Appl. Mater. Interfaces* **2023**, *15*, 9494−9507.

3. Wang, J.; Chen, T.; Jeon, M.; Oppenheim, J. J.; Tan, B.; Kim, J.; Dinca, M. Superior Charge Transport in Ni-Diamine Conductive MOFs. *J. Am. Chem. Soc.* **2024**, *146* (29), 20500–20507.

4. Chen, T.; Dou, J.-H.; Yang, L.; Sun, C.; Libretto, N. J.; Skorupskii, G.; Miller, J. T.; Dinca, M. Continuous Electrical Conductivity Variation in M_3(Hexaiminotriphenylene)$_2$ (M=Co, Ni, Cu) MOF Allloys. *J. Am. Chem. Soc.* **2020**, *142* (28), 12367–12373.

5. Gittins, J. W.; Balhatchet, C. J.; Chen, Y.; Liu, C.; Madden, D. G.; Britto, S.; Golomb, M. J.; Walsh, A.; Fairen-Jimenez, D.; Dutton, S. E.; Forse, A. C., Insights into the Electric Double-Layer Capacitance of Two-Dimensional Electrically Conductive Metal-Organic Frameworks. *J. Mater. Chem. A* **2021**, *9* (29), 16006−16015.

6. Liu, Y.; Yao, H.; Zhang, H.; Ma, C.; Guo, J.; Fan, Y.; Chen, H.; Wu, X.; Yang, L.; Huang, X.; Chen, T.; Ji, Q.; Yao, Z.-F.; Li, J.; Dou, J.-H., Asymmetrical Substitution Manipulates Stacking Modes in 2D Conductive MOF Crystals. *J. Am. Chem. Soc.* **2025**, *147* (52), 48127−48135.

7. Ko, M.; Aykanat, A.; Smith, M. K.; Mirica, K. A. Drawing Sensors with Ball-Milled Blends of Metal-Organic Frameworks and Graphite. *Sensors* **2017**, *17* (10), 2192.

8. Pham, H. T. B.; Choi, J. Y.; Huang, S.; Wang, X.; Claman, A.; Stodolka, M.; Yazdi, S.; Sharma, S.; Zhang, W.; Park, J., Imparting Functionality and Enhanced Surface Area to a 2D Electrically Conductive MOF via Macrocyclic Linker. *J. Am. Chem. Soc.* **2022**, *144* (23), 10615−10621.

9. Sheberla, D.; Sun, L.; Blood-Forsythe, M. A.; Er, S.; Wade C. R.; Brozek, C. K.; Aspuru-Guzik, A.; Dinca, M. High Electrical Conductivity in Ni_3(2,3,6,7,10,11-hexaiminotriphenylene)$_2$, a Semiconducting Metal-Organic Graphene Analogue. *J. Am. Chem. Soc.* **2014**, *136* (25), 8859−8862.

10. Huang, X.; Sheng, P.; Tu, Z.; Zhang, F.; Wang, J.; Geng, H.; Zou, Y.; Di, C.-A.; Yi, Y.; Sun, Y.; Xu, W.; Zhu, D. A Two-Dimensional π-d Conjugated Coordination Polymer with Extremely High Electrical Conductivity and Ambipolar Transport Behavior. *Nat. Commun.* **2015**, 6, 7408.

11. Dong, R.; Zhang, Z.; Trana, D. C.; Zhou, S.; Wang, M.; Adler, P.; Liao, Z.; Liu, F.; Sun, Y.; Shi, W.; Zhang, Z.; Zschech, E.; Mannsfeld, S. C. B.; Felser, C.; Feng, X. A Coronene-Based Semiconducting Two-Dimensional Metal-Organic Framework with Ferromagnetic Behavior. *Nat. Commun.* **2018**, 9, 2637.

12. Sporrer, L.; Zhou, G.; Wang, M.; Balos, V.; Revuelta, S.; Jastrzembski, K.; Loffler, M.; Petkov, P.; Heine, T.; Kuc, A.; Canovas, E.; Huang, Z.; Feng, X.; Dong, R. Near IR Bandgap

Semiconducting 2D Conjugated Metal-Organic Framework with Rhombic Lattice and High Mobility. *Angew. Chem.* Int. Ed. **2023**, *62* (25), e202300186.

13. Kambe, T.; Sakamoto, R.; Hoshiko, K.; Takada, K.; Miyachi, M.; Ryu, J.-H.; Sasaki, S.; Kim, J.; Nakazato, K.; Takata, M.; Nishihara, H. π-Conjugated Nickel Bis(dithiolene) Complex Nnaosheet. *J. Am. Chem. Soc.* **2013**, *135* (7), 2462−2465.

14. Kambe, T.; Sakamota, R.; Kusamoto, T.; Pal, T.; Fukui, N.; Hoshiko, K.; Shimojima, T.; Wang, Z.; Hirahara, T.; Ishizaka, K.; Hasegawa, S.; Liu, F.; Nishihara, H. Redox Control and High Conductivity of Nickel Bis(dithiolene) Complex π-Nanosheet: A Potential Organic Two-Dimensional Topological Insulator. *J. Am. Chem. Soc.* **2014**, *136* (41), 14357−14360.

15. Apostol, P.; Gali, S. M.; Su, A.; Tie, D.; Zhang, Y.; Pal, S.; Lin, X.; Bakuru, V. R.; Rambabu, D.; Beljonne, D.; Dincă, M.; Vlad, A., Controlling Charge Transport in 2D Conductive MOFs─The Role of Nitrogen-Rich Ligands and Chemical Functionality. *J. Am. Chem. Soc.* **2023**, *145* (45), 24669−24677.

16. Lu, Y., Zhang, Y., Yang, CY. *et al.* Precise Tuning of Interlayer Electronic Coupling in Layered Conductive Metal-Organic Frameworks. *Nat. Commun.* **2022***, 13*, 7240.

17. Noh, H.-J.; Cline, E.; Pennington, D. L.; Lin, H.-Y. G.; Hendon, C. H.; Mirica, K. A., Tuning the Structure–Property Relationships of Metallophthalocyanine-Based Two-Dimensional Conductive Metal–Organic Frameworks with Different Metal Linkages. *J. Am. Chem. Soc.* **2025**, *147* (10), 8240−8249.

18. Yi, J.-D.; So, D.-H.; Xie, R.; Yin, Q.; Zhang, M.-D.; Wu, Q.; Chai, G.-L.; Huang, Y.-B.; Cao, R. Conductive Two-Dimensional Phthalocyanine-based Metal-Organic Framework Nanosheets for Efficient Electroreduction of CO_2. *Angew. Chem.* Int. Ed. **2021**, *60* (31), 17108–17114.

19. Meng, Z.; Aykanat, A.; Mirica, K. A., Welding Metallophthalocyanines into Bimetallic Molecular Meshes for Ultrasensitive, Low-Power Chemiresistive Detection of Gases. *J. Am. Chem. Soc.* **2019**, *141* (5), 2046−2053.

20. Meng, Z.; Luo, J.; Li, W.; Mirica, K. A., Hierarchical Tuning of the Performance of Electrochemical Carbon Dioxide Reduction Using Conductive Two-Dimensional Metallophthalocyanine Based Metal-Organic Frameworks. *J. Am. Chem. Soc.* **2020**, *142* (52), 21656−21669.

21. Park, J.; Hinckley, A. C.; Huang, Z.; Yakovenko, A. A.; Lee, M.; Chen, S.; Zou, X.; Bao, Z. Synthetic Routes for a 2D Semiconductive Copper Hexahydroybenzene Metal-Organic Framework. *J. Am. Chem. Soc.* **2018**, *140* (44), 14533−14537.

22. Noh, H.-K.; Pennington, D. L.; Seo, J.-M.; Cline, E.; Benedetto, G.; Baek, J.-B.; Hendon, C. H.; Mirica, K. A. Reversible and Ultrasensitive Detection of Nitric Oxide Using a Conductive Two-Dimensional Metal-Organic Framework. *Angew. Chem. Int. Ed.* **2025**, *64*, e202419869.

23. Noh, H.-K.; Qing, H.; Wang, P.; Li, W.; Mirica, K. A. Incorporating Redox-Active Hexaazatrinapthylene into a 2D Conductive Metal-Organic Framework for Robust Sodium-Ion Batteries. *Angew. Chem. Int. Ed.* **2026**, *65*, e16381.

24. Yang, M.; Zhang, Y.; Zhu, R.; Tan, J.; Liu, J.; Zhang, W.; Zhou, M.; Meng, Z. Two-Dimensional Conjugated Metal-Organic Frameworks with a Ring-in-Ring Topology and High Electrical Conductance. *Angew. Chem. Int. Ed.* **2024**, *63*, e20240533.

25. Qing, H.; Diamond, B. G.; Chan, J. Y. M.; Hendon, C. H.; Li, W.; Mirica, K. A. Isoreticular Modulation of Electrical Conduction and Magnetic Properties in Semiconducting Lanthanide-based Based Metal−Organic Frameworks. *Angew. Chem. Int. Ed.* **2026**, *65* (21), e8403106.

26. Su, A. Y.; Oppenheim, J. J.; Dinca, M. Dual Proton-Electron Conductivity in 2D Azaborine Metal-organic Frameworks. *J. Am. Chem. Soc.* **2025**, *147* (40), 36837–36842.

27. Su, J.; He, W.; Li, X. M.; Sun, L.; Wang, H. Y.; Lan, Y. Q.; Ding, M.; Zuo, J. L. High Electrical Conductivity in a 2D MOF with Intrinsic Superprotonic Conduction and Interfacial Pseudo-Capacitance. *Matter* **2020**, *2* (3), 711–722.

28. Rambabu, D.; Goossens, T.; Bakuru, V. R.; Apostol, P.; Mairesse, F.; Steenhaut, T.; Beaujean, P.; Mondal, S. K.; Guo, X.; Zhang, Y.; Pal, S.; Markowski, R.; Lin, X.; Xu, P.; Chanteux, G.; Kachmar, A.; Tie, D.; Ramackers, A.; Frano, V.; Robeyns, K.; Kumar Maji, T.; Filinchuk, Y.; Champagne, B.; Vlad, A. Mixed Proton-Electron Conductivity in a Dynamic 3D Metal-Organic Framework. *Chem* **2025**, *11*, 102590.

29. Ribeiro, C.; Tan, B.; Figueira, F.; Mendes, R. F.; Calbo, J.; Valente, G.; Escamilla, P.; Paz, F. A. A.; Rocha, J.; Dincă, M.; Souto, M. Mixed Ionic and Electronic Conductivity in a Tetrathiafulvalene-Phosphonate Metal-Organic Framework. *J. Am. Chem. Soc.* **2025**, *147*, 63−68.

30. Choi, J. Y.; Stodolka, M.; Kim, N.; Pham, H. T. B.; Check, B.; Park, J. 2D Conjugated Metal-Organic Framework as a Proton-Electron Dual Conductor. *Chem* **2023**, *9* (1), 143–153.

31. Park, M.; Ju, H.; Oh, J.; Park, K.; Lim, H.; Yoon, S. M.; Song, I. Proton-Electron Coupling and Mixed Conductivity in a Hydrogen-Bonded Coordination Polymer. *Nat. Comm.* **2025**, *16* (1), 1–12.

32. He, X.-L.; Shao, B.; Huang, R.-K.; Dong, M.; Tong, Y.-Q.; Luo, Y.; Meng, T.; Yang, F.-J.; Zhang, Z.; Huang, J.; He, X.-L.; Shao, B.; Dong, M.; Luo, Y.; Meng, T.; Huang, J.; Tong, Y.-Q.; Zhang, Z.; Huang, R.-K.; Yang, F.-J. A Mixed Protonic–Electronic Conductor Base on the Host-Guest Architecture of 2D Metal–Organic Layers and Inorganic Layers. *Adv. Sci.* **2023**, *10* (17), 2205944.

33. Saha, R.; Sharma, A.; Siddiqui, A. I.; Benmansour, S.; Ortega-Castro, J.; Frontera, A.; Mondal, B.; Lah, M. S.; Gomez Garcia, C. J., Simultaneous Electron and Proton Conduction in a Stable Metal Organic Material with Highly Selective Electrocatalytic Oxygen Reduction Reaction to Water. *Chem. Sci.* **2025**, *16* (21), 9501−9508.

34. Jo, Y. M.; Kim, D. H.; Wang, J.; Oppenheim, J. J.; Dinca, M., Humidity-Mediated Dual Ionic-Electronic Conductivity Enables High Sensitivity in MOF Chemiresistors. *J. Am. Chem. Soc.* **2024**, *146* (29), 20213−20220.

35. Bashiri, R.; Lawson, P. S.; He, S.; Nanayakkara, S.; Kim, K.; Barnett, N. S.; Stavila, V.; El Gabaly, F.; Lee, J.; Ayars, E.; So, M. C., Discovery of Dual Ion-Electron Conductivity of Metal–Organic Frameworks via Machine Learning-Guided Experimentation. *Chem. Mater.* **2025**, *37* (3), 1143−1153.
